# Structure‐Based Design of a Macrocyclic PROTAC

**DOI:** 10.1002/anie.201914396

**Published:** 2019-12-12

**Authors:** Andrea Testa, Scott J. Hughes, Xavier Lucas, Jane E. Wright, Alessio Ciulli

**Affiliations:** ^1^ Division of Biological Chemistry and Drug Discovery School of Life Sciences University of Dundee Dow Street Dundee DD1 5EH Scotland UK; ^2^ Current address: Roche Pharma Research and Early Development Roche Innovation Center Basel F. Hoffmann-La Roche Ltd. Grenzacherstrasse 124 CH-4070 Basel Switzerland

**Keywords:** drug design, macrocycles, protein structures, protein–protein interactions, proteolysis-targeting chimeras (PROTACs)

## Abstract

Constraining a molecule in its bioactive conformation via macrocyclization represents an attractive strategy to rationally design functional chemical probes. While this approach has been applied to enzyme inhibitors or receptor antagonists, to date it remains unprecedented for bifunctional molecules that bring proteins together, such as PROTAC degraders. Herein, we report the design and synthesis of a macrocyclic PROTAC by adding a cyclizing linker to the BET degrader MZ1. A co‐crystal structure of macroPROTAC‐1 bound in a ternary complex with VHL and the second bromodomain of Brd4 validated the rational design. Biophysical studies revealed enhanced discrimination between the second and the first bromodomains of BET proteins. Despite a 12‐fold loss of binary binding affinity for Brd4, macroPROTAC‐1 exhibited cellular activity comparable to MZ1. Our findings support macrocyclization as an advantageous strategy to enhance PROTAC degradation potency and selectivity between homologous targets.

## Graphical abstract

**Closing the circle**: A macrocyclic PROTAC has been designed and synthesized by adding a cyclizing linker to MZ1, a PROTAC targeting the bromodomain of the BET protein, Brd4. A co‐crystal structure of macroPROTAC‐1 bound in a ternary complex with E3 ligase VHL and the second bromodomain of Brd4 validated the rational design. This macrocyclization strategy enhanced the target specificity and cellular activity of the PROTAC.

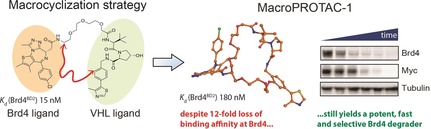

## Introduction

Targeted protein degradation is a powerful new modality of chemical biology and drug discovery.^1^, ^2^ PROTACs are bifunctional molecules composed of a ligand for a target protein of interest, and a ligand for an E3 ubiquitin ligase, joined by a flexible linker. Recruitment of a protein close to an E3 ligase hijacks the ligase catalytic activity, leading to the tagging of the target protein by ubiquitination and subsequent proteasomal degradation.^3^, ^4^, ^5^ PROTACs are emerging as attractive chemical probes and therapeutics, defined by a catalytic and sub‐stoichiometric rather than occupancy‐based mode of action, leading to an extended duration of action.^6^, ^7^, ^8^, ^9^ Due to their different mechanism of action via formation of a ternary complex species, PROTACs have the potential to pharmacologically differentiate from inhibitors and to more effectively impact biology and disease, including targeting proteins that have proven intractable to other approaches.

Structural and biophysical investigation of ternary complexes from our laboratory revealed that proximity‐induced formation of de novo interactions within the ternary complex are an important feature of the PROTAC mode of action.^10^ Neomorphic interactions include protein–protein interactions (PPIs) between the ligase and the target, which can lead to the formation of cooperative and stable ternary complexes.^11^ These characteristics of the ternary complex, albeit not strictly essential for PROTAC‐mediated protein degradation,^12^, ^13^ are important because they can increase the population of the complex (the key Michaelis–Menten species in the PROTAC‐mediated catalytic process) and contribute to enhancing its thermodynamic stability and kinetic dissociative half‐life.^10^, ^14^, ^15^ These features in turn enhance the catalytic efficiency of the process, leading to greater levels of target ubiquitination, and consequently faster and more prolonged target degradation.^15^, ^16^ Because the PROTAC‐induced PPIs involve less‐conserved regions outside of the ligand‐binding pocket, PROTACs made of promiscuous or non‐selective target ligands can discriminate between highly homologous target proteins in a way not achievable with the parent inhibitors alone.^7^, ^10^, ^13^, ^15^, ^17^, ^18^

Knowledge of the PROTAC binding mode in a ternary complex, ideally from co‐crystal structures, provides an opportunity to rationally design compounds to better fit and stabilize the newly created interface within the ternary complex, using structure‐based drug design.^10^, ^13^, ^19^, ^20^ Our first crystal structure of the PROTAC MZ1 in complex with the E3 ligase VHL and its target, the second bromodomain (BD2) of the bromodomain and extra‐terminal motif (BET) protein, Brd4, revealed that the two ligand moieties of MZ1 (the VHL ligand, VH032, and the BET inhibitor, JQ1) lay in close spatial proximity within the ternary complexes.^10^ We thus imagined that a macrocyclic PROTAC could be designed based on the crystal structure as a strategy to lock the PROTAC conformation in the bound state. Macrocyclization is expected to reduce the energetic penalty to adopt the bound state through conformational restriction.^21^, ^22^ As such, macrocycles provide privileged scaffolds to target PPIs and protein surfaces, amongst other challenging targets.^23^, ^24^, ^25^ For example, the archetypal macrolide natural products cyclosporine and rapamycin are macrocyclic molecules that act as molecular glues to induce the formation of a ternary complex.^26^, ^27^ To our knowledge, however, the use of macrocylization as a strategy for PROTAC design, or more generally to bias recruitment of proteins together, remains unprecedented. Herein, we provide first proof‐of‐concept validation of the approach with a macrocyclic PROTAC. The design strategy was aided by computational calculations and molecular dynamics (MD) simulations based on the VHL:MZ1:BRD4^BD2^ ternary‐complex crystal structure. Macrocyclization was achieved by adding a second linker to “close a circle” between the two ligand moieties of MZ1. Using isothermal titration calorimetry (ITC), fluorescence polarization (FP), and X‐ray crystallography, we show that macroPROTAC‐1 better discriminates between the recruitment of the second and the first BET bromodomains to VHL. Despite a greater than 10‐fold loss in binary binding affinity for Brd4, macroPROTAC‐1 exhibits rapid and potent intracellular degradation of Brd4, and cytotoxicity in BET‐sensitive cancer cell lines that are comparable to MZ1.

## Results and Discussion

**Figure 1:**
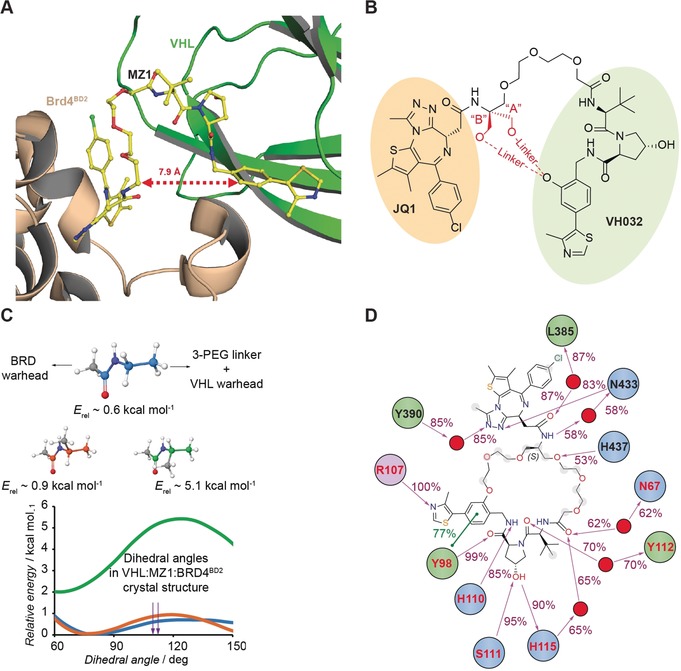
Structure‐based design of macrocyclic PROTAC **1**. A) Crystal structure of MZ1 in complex with Brd4^BD2^ and VHL‐EloB‐EloC shows MZ1 in a horseshoe shape, suggesting potential for cyclization (PDB code 5T35).^10^ B) The two cyclization vectors “A” and “B” investigated computationally in this study. In black the chemical structure of MZ1, in red the additional linker. C) Torsional energy profiling along the alkylamide dihedral of N‐ethylacetamide (blue) and N‐isopropylacetamide (orange and green), as surrogates of the chemical environment in the linker of MZ1 and its macrocyclic derivatives. D) Chemical structure of **1** and its non‐covalent interactions with VHL (residues in red), BRD4^BD2^ (residues in black) and water (red spheres), as observed during the last 50 ns of a 200 ns MD simulation of the VHL:**1**:BRD4^BD2^ ternary complex. The percentage of time spent in the interaction is shown. Charged, polar, and hydrophobic amino acids are colored in purple, blue, and green, respectively, and the solvent‐exposure of atoms in **1** is highlighted in a gray shadow.

Inspired and guided by the crystal structure of the ternary complex between our BET degrader MZ1, VHL, and Brd4^BD2^,^10^ we designed a series of macrocyclic PROTACs with the aim to lock the PROTAC in the bound conformation (Figure 1 A). We hypothesized that macrocyclization would increase the energetic bias towards the productive PROTAC ternary complex, relative to either non‐productive ternary complexes (namely, those that do not lead to target ubiquitination), binary complexes, or free in solution. Our approach to cyclize MZ1 relied upon identifying a suitable vector and linker that would retain the binding mode of the linear compound and at the same time maintain the induced PPIs that contribute to forming a stable and cooperative ternary complex that underpins the preferential degradation of BRD4.^10^, ^15^ Two vectors connecting a phenolic group on the VHL ligand to the same carbon of the first PEG unit of the MZ1 linker were explored (vectors A and B, Figure 1 B).

We first studied computationally the potential energy strain introduced by alkylation in vectors A and B in the JQ1‐amide (Figure 1 B), using the model compound *N*‐ethylacetamide (Figure 1 C).^28^ We observed that the alkylamide torsion angles in the crystal structure of MZ1 (110° and 112°)^10^ are low‐energy states in the surrogate *N*‐ethylacetamide. Alkylation at vector A would force the amide to an eclipsed conformer, for which we found an energy penalty of approximately 5 kcal mol^−1^. This modification would prevent the adoption of the crystallographic pose of the MZ1 linker, thereby disrupting the binding mode. We therefore deemed a derivatization from vector B to be more suitable. Molecular modelling studies were performed on derivatives **1** and **2** (Supporting Information, Figure S1 A) cyclized from vector B with a linker comprising 3 and 2 PEG units, respectively. The modelling indicated that a 2‐PEG linker, as in compound **2** (Supporting Information, Figure S1 A), would be too short to cover the distance between the two attachment points and would impair binding of the MZ1 core (Supporting Information, Figure S1 B). In contrast, a linker comprising 3 PEG units (compound **1,** Supporting Information, Figure S1 A) could be well accommodated in the cavity formed between the proteins (Supporting Information, Figure S1 C). We next performed molecular dynamics (MD) simulations of **1** bound to VHL and BRD4^BD2^ in order to investigate the behavior of the ternary system in solution. The ternary complex remained stable throughout the simulation, with retention of the binding mode for the MZ1 portion of **1** and conservation of the interprotein and protein–PROTAC contacts observed in the VHL:MZ1:BRD4^BD2^ structure (Supporting Information, Figures S2 and S3). Crucially, the simulations suggested that macrocyclization was compatible with key polar interactions at each end of the new linker, including the water‐mediated interaction of the JQ1‐amide with N433^BRD4(BD2)^, the H‐bond of the oxygen atom in the first PEG unit in MZ1 with H437^BRD4(BD2)^, and the H‐bond with Y98^VHL^ (Figure 1 D). Only a very modest shortening between the attachment points was observed in the macrocycle (7.7±0.3 Å, Supporting Information, Figure S2 A) compared to MZ1 (7.9 Å and 8.2 Å in the two instances of the asymmetric unit),^10^ as well as the formation of a stable water network at the proteins–PROTAC interface contributed by polar interactions with each partner (Supporting Information, Figure S3 B). Thus, we selected **1** as the target macrocyclic derivative of MZ1 for synthesis.

**Scheme 1:**
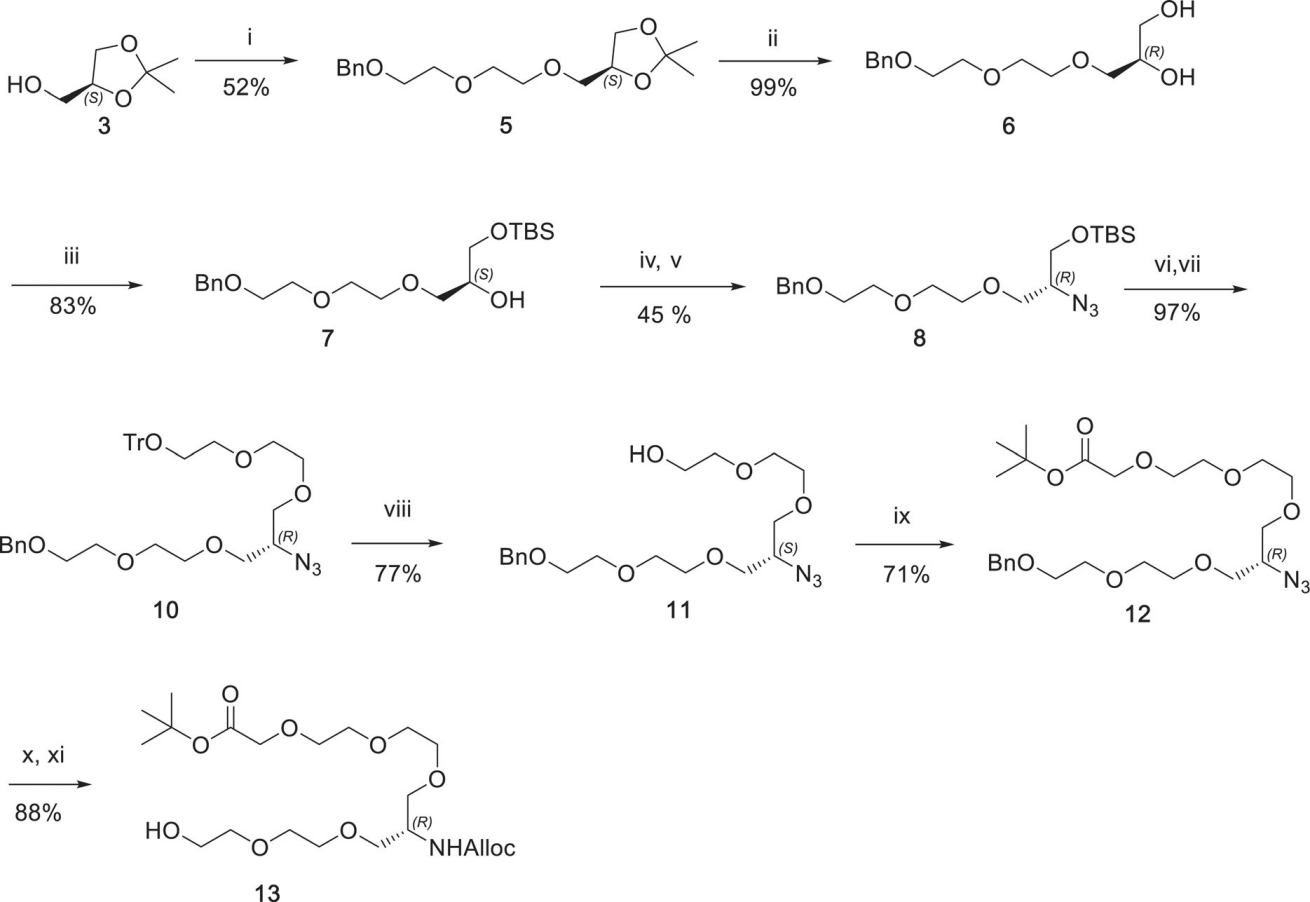
Synthesis of trifunctional linker **13**. i) 2‐(2‐(benzyloxy)ethoxy)ethyl 4‐methylbenzenesulfonate (**4**), KOH, TBAI, Dioxane; ii) 80 % AcOH in H_2_O; iii) TBSCl, TEA, DMAP, DCM; iv) MsCl, Py; v) NaN_3_, DMF; vi) TBAF, THF; vii) 2‐(2‐(trityloxy)ethoxy)ethyl methanesulfonate (**9**), KOH, TBAI, Dioxane; viii) 10 % TFA/TIPS in DCM; ix) tert‐butyl 2‐bromoacetate, TBAB, 37 % NaOH in H_2_O; x) H_2_, Pd/C, AcOH, EtOH; *N*‐(allyloxycarbonyloxy)succinimide, NaHCO_3_, dioxane/H_2_O (2:1).

In order to synthetically achieve our macrocyclic PROTAC **1**, a bespoke linker able to connect the BET ligand to the two different attachment points on the VHL ligand needed to be designed. We envisaged a retrosynthetic strategy based on the *O*‐alkylation of the VHL ligand with a trifunctional PEG linker bearing a carboxylic acid for the macrolactamization and a protected amine for coupling to JQ1. The new stereocenter generated from the formal alkylation of the first ethylene glycol unit of MZ1 could be easily set by choosing the correct chiral glycerol equivalent. The synthesis of the trifunctional linker is detailed in Scheme 1 and started from commercially available (*S*)‐(+)‐1,2‐isopropylideneglycerol **3** and 2‐(2‐(benzyloxy)ethoxy)ethyl 4‐methylbenzenesulfonate **4**. Alkylation was performed in solid–liquid phase transfer catalysis (S‐L PTC) conditions using potassium hydroxide as a base and tetrabutylammonium iodide as catalyst and afforded compound **5** in 52 % yield. The acetonide deprotection was achieved in aqueous acetic acid to obtain the diol **6** in quantitative yield. Selective primary alcohol protection with *tert*‐butyl dimethyl silyl chloride afforded the TBS ether **7**, which underwent functional‐group interconversion via mesylate to obtain the azide **8**. Subsequent deprotection of the silyl ether and alkylation with mesylate **9** led to the trityl‐protected compound **10** in excellent yield. Removal of the trityl group was achieved with diluted TFA and triisopropyl silane (10 % in DCM) to afford alcohol **11**. Reaction with *tert*‐butyl bromoacetate in PTC conditions led to ester **12** in 71 % yield. Hydrogenative palladium‐catalyzed debenzylation and azide reduction, followed by Alloc protection afforded the key trifunctional linker **13**.

**Scheme 2:**
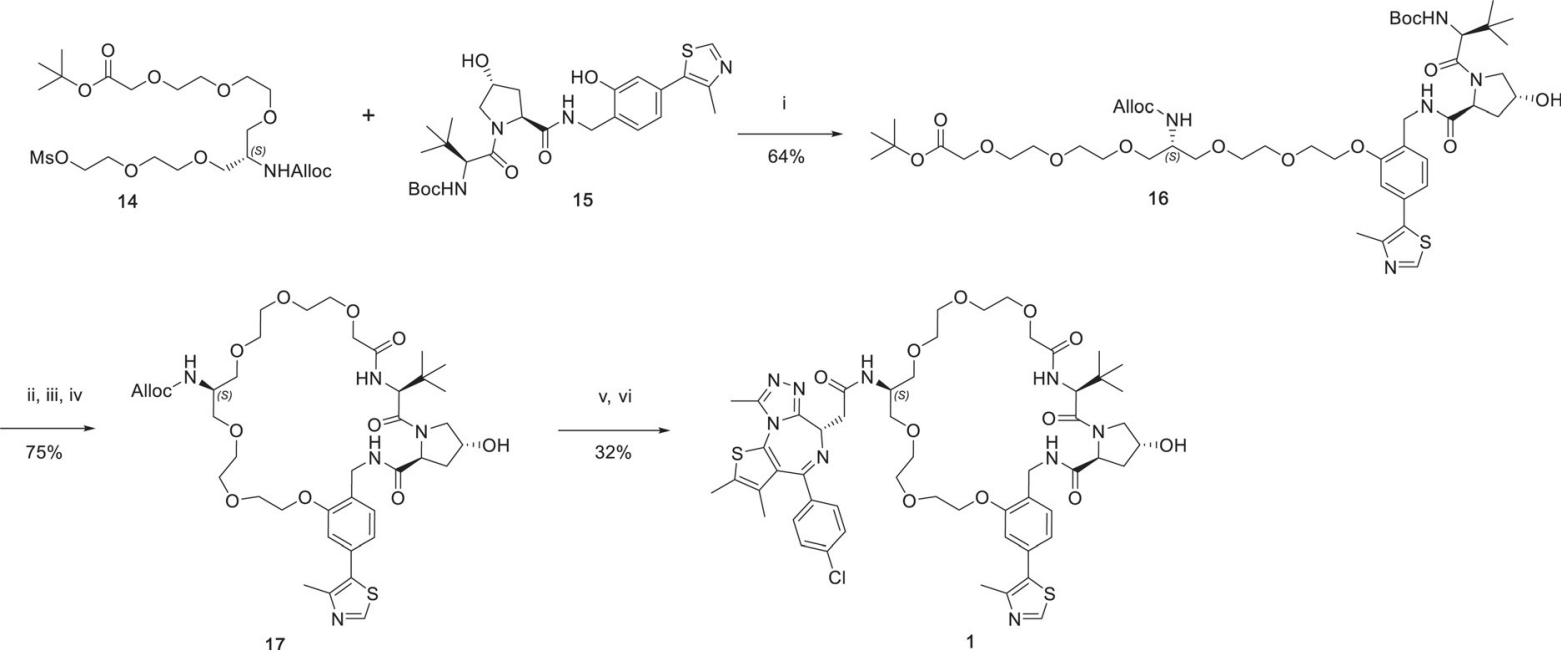
Macrolactamization and completion of the synthesis of macroPROTAC‐1. i) K_2_CO_3_, DMF; ii) TFA, DCM; iii) HCl, H_2_O; iv) HATU, DIPEA, DMF; v) Pd(PPh_3_)_4_, PhSiH_3_, THF; vi) JQ1‐COOH, COMU, DIPEA, DMF.

With the trifunctional linker in hand, we next proceeded to alkylate the Boc‐protected VHL ligand **15**. The mesylate **14** (obtained from **13** and mesyl chloride) was reacted with **15** and potassium carbonate in DMF to give compound **16** in 64 % yield. The latter was treated with TFA to simultaneously deprotect the *tert*‐butyl ester and the Boc protecting group. After exchanging the trifluoroacetate counterion with hydrochloride by freeze drying from a diluted HCl solution, macrolactamization was performed with HATU in high dilution (1.7 mm in DCM) to afford compound **17** in 75 % yield. Alloc deprotection was achieved with tetrakis(triphenylphosphine)palladium and an excess of phenylsilane. Finally, amide coupling with JQ1‐COOH in the presence of COMU and DIPEA afforded compound **1** in 32 % isolated yield from **17** (Scheme 2).

**Figure 2:**
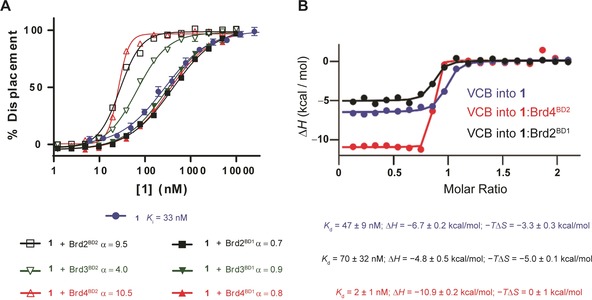
Binding affinity and cooperativity of ternary complex formation. A) Dose‐dependent displacement of a fluorescent HIF‐1α peptide by **1** alone or **1** in complex with a BET bromodomain, measured by fluorescence polarization. Cooperative binding is observed for all the BD2s while slightly negatively cooperative binding is observed for the BD1s. B) Representative inverse ITC titrations of VCB into **1** and VCB into **1**:Brd2^BD1^ or **1**:Brd4^BD2^ showing cooperative binding in the case of Brd4^BD2^ and negatively cooperative binding in the case of Brd2^BD1^.

To assess the extent to which macrocyclization impacted on the formation of PROTAC ternary complexes between VHL and BET bromodomains, we next turned to biophysical ternary complex assays. First, we performed a competitive fluorescence polarization (FP) assay.^15^ In this assay, a fluorescent HIF‐1α peptide probe bound to VHL is displaced in a dose‐responsive fashion by either PROTAC alone or PROTAC pre‐incubated with individual BET bromodomains. Positive cooperativity (α) would result in a left‐shift of the curve (higher binding affinity) in the presence of the bromodomain. Compound **1** (herein referred to as macroPROTAC‐1) retained high binding affinity to VHL (*K*_i_=33 nm, Figure 2 A). We observed cooperative formation of ternary complexes with the BD2s of Brd4, Brd2 and, to a lesser extent, Brd3 (*α*=10.5, 9.5, and 4.0, respectively). In contrast, no cooperativity was observed with the first bromodomain (BD1) of Brd3, Brd4, and Brd2 (*α*=0.9, 0.8, and 0.7, respectively). This contrasts with MZ1, which could still form cooperative complexes with all the target bromodomains,^10^, ^15^ and suggests that macroPROTAC‐1 better differentiates the second bromodomains versus the first bromodomains of the BET proteins compared to MZ1.

To gain more insights on the effect of cyclization on the thermodynamics of ternary‐complex formation, the binding affinity of macroPROTAC‐1 for VHL and selected BET bromodomains was studied by ITC (Supporting Information, Figure S4). For these experiments, among the six BET bromodomains, we selected Brd2^BD1^ and Brd4^BD2^ as they respectively formed the least cooperative and most cooperative ternary complexes, according to our FP data. MacroPROTAC‐1 bound to VHL with *K*_d_=47±9 nm and Δ*H* of −6.7±0.2 kcal mol^−1^ (Figure 2 B), comparable to MZ1 (*K*_d_=66±6 nm and Δ*H* of −7.7±0.3 kcal mol^−1^).^10^ Interestingly, much weaker binary binding affinities were detected for the bromodomains (Brd4^BD2^ *K*_d_=180 nm, compared with 15 nm for MZ1; Brd2^BD1^ *K*_d_=740 nm, compared with 62 nm for MZ1, Table 1), corresponding to a 12‐fold loss of binary affinity compared to MZ1 in each case.^10^ Thermodynamics of formation of ternary complexes VHL:**1**:Brd2^BD1^ and VHL:**1**:Brd4^BD2^ revealed high positive cooperativity of VHL–Brd4^BD2^ (*α*=20, compared with 17.6 for MZ1) and a negatively cooperative complex with VHL–Brd2^BD1^ (*α*=0.7, compared with 2.9 for MZ1, Figure 2 B and Table 1). Together, the biophysical data is consistent with a better discrimination between the highly homologous BET bromodomains when using macroPROTAC‐1 compared to its non‐cyclic progenitor.

**Table 1:** Thermodynamic parameters of formation of binary and ternary complexes between **1** or MZ1, and VHL–ElonginC–ElonginB (VCB), Brd2^BD1^, and Brd4^BD2^. The reported values are the mean±standard deviation from independent measurements. For titrations of MZ1, the data is taken from ref. 10.

Protein in syringe	Species in cell	*K*_d_ [nm]	Δ*H* [kcal mol^−1^]	Δ*G* [kcal mol^−1^]	−*T*Δ*S* [kcal mol^−1^]	α	Total Δ*G* [kcal mol^−1^]	No. of replicates
Brd2^BD1^	**1**	743±202	−9.6±0.6	−8.4±0.2	1.2±0.7	–	–	2
Brd4^BD2^	**1**	180±42	−6.25±0.17	−9.2±0.2	−2.7±0.3	–	–	2
VCB	**1**	47±9	−6.7±0.2	−10.0±0.1	−3.3±0.3	–	–	3
VCB	**1**: Brd2^BD1^	70±32	−4.8±0.5	−9.9±0.4	−5.0±0.1	0.7	−18.3±0.4	2
VCB	**1**: Brd4^BD2^	2±1	−10.9±0.2	−11.9±0.3	−1.0±0.4	20	−21.1±0.4	2
Brd2^BD1^	MZ1	62±6	−12.8±0.7	−9.84±0.06	3.0±0.8	–	–	2
Brd4^BD2^	MZ1	15±1	−10.9±0.4	−10.68±0.04	0.2±0.4	–	–	2
VCB	MZ1	66±6	−7.7±0.3	−9.81±0.05	−2.1±0.3	–	–	8
VCB	MZ1: Brd2^BD1^	24±8	−7.3±0.2	−10.4±0.2	−3.1±0.4	2.9	−20.3±0.2	2
VCB	MZ1: Brd4^BD2^	3.7±0.7	−8.9±0.1	−11.5±0.1	−2.6±0.2	17.6	−22.2±0.2	2

**Figure 3:**
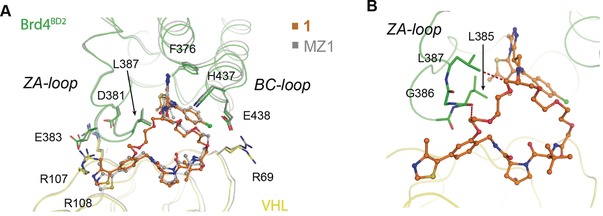
Crystal structure of ternary complex VHL:**1**:Brd4^BD2^. A) Superposition of VHL:**1**:Brd4^BD2^ (green and yellow) and VHL:MZ1:Brd4^BD2^ ternary complex (PDB 5T35; grey) highlighting the key protein‐protein interactions between VHL and Brd4^BD2^. B) Residues in the ZA‐loop of Brd4^BD2^ (Leu385, Gly386 and Leu387) that are in close proximity to the additional linker on **1**. Red dashes indicate atom pairs that are ≤3.5 Å apart.

To validate the binding mode and deepen understanding of the molecular basis for the biophysical properties of macroPROTAC‐1, we next co‐crystallized VHL:macroPROTAC‐1:Brd4^BD2^ and solved the structure of the ternary complex at a resolution of 3.5 Å (Figure 3 and Supporting Information, Figure S5). The structure superposes well with the ternary complex VHL:MZ1:Brd4^BD2^ (Cα RMSD=0.6 Å) and recapitulates the major PPIs between VHL and Brd4^BD2^. Conserved contacts include the previously reported electrostatic interactions between Arg107^VHL^, Arg108^VHL^, D381^Brd4(BD2)^ and E383^Brd4(BD2)^; and the stack between the canonical WPF shelf of Brd4^BD2^ and Pro71 of VHL (Figure 3 A). Collectively, these interactions result in a buried surface area (BSA) between the two proteins of 681 Å^2^. The MZ1‐portion of macroPROTAC‐1 binds in an identical S‐shaped conformation to the uncyclized PROTAC, retaining the H‐bond between His437^Brd4(BD2)^ and an oxygen atom on the PEG‐3 linker. The cyclizing part of the linker optimally fills an additional cavity created at the interface of the two proteins next to the ZA‐loop of Brd4^BD2^ (Figure 3 A and Supporting Information, Figure S6), which is in good agreement with the MD simulations (Figure 1 D and Supporting Information, Figure S2). The BSA at the macroPROTAC‐1:VHL and macroPROTAC‐1:Brd4^BD2^ interfaces are 961 and 1064 Å^2^, respectively, which brings the total BSA to 2686 Å^2^. Taken together, these findings could explain the high cooperativity of VHL:macroPROTAC‐1:Brd4^BD2^. Closer examination of the additional linker revealed potential clashes with the ZA‐loop, which could explain the loss in binding affinity with the BET bromodomains. Within the ZA‐loop, the side chain of Leu387, as well as the carbonyl oxygens of both Gly386 and Leu385, are less than 3.5 Å from the newly added linker. Interestingly, the clash with Leu387 is similar to that exploited in our bump‐and‐hole study for the same residue (Figure 3 B).^29^, ^30^ The enhanced discrimination between BD1 and BD2 could potentially be attributed to differences in the ZA‐loop of BD1s compared to BD2s. Sequence alignment of the six bromodomains revealed an additional proline (Pro397) in BD1 which could limit the ability of the BD1s to accommodate the extra linker present in macroPROTAC‐1 (Supporting Information, Figure S7).

**Figure 4:**
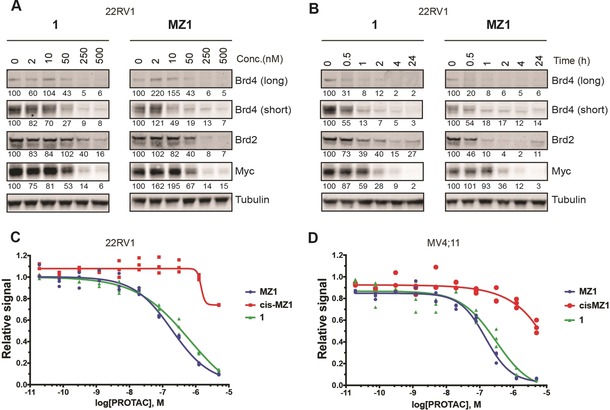
Cellular activity of macroPROTAC‐1. A) 22RV1 cells were treated with different concentrations of **1** or MZ1 for 4 h. B) 22RV1 cells were treated with 500 nm of **1** or MZ1 for different times. In (A) and (B), protein levels were analyzed by western blotting using the corresponding antibodies after SDS‐PAGE. The quantity of protein in each band is shown as a percentage underneath. Each band was normalized relative to tubulin and the untreated condition. C,D) Antiproliferative activity of **1**, MZ1, and cisMZ1. 22RV1 (C) and MV4;11 (D) cells were treated with compounds for 72 and 48 h, respectively, prior to quantitation of cell proliferation using the CellTiter‐Glo luminescent cell viability assay.

We next investigated the cellular activities of macroPROTAC‐1. First, HeLa cells were treated with macroPROTAC‐1 or MZ1 for 4 h or 18 h before cell lysis and western blotting (Supporting Information, Figure S8). MacroPROTAC‐1, like MZ1, was able to induce potent and rapid degradation of Brd4 (both short and long isoforms) with a DC_50_ between 25 and 125 nm, while degradation of Brd2 and Brd3 occurred only at higher concentrations, with a DC_50_ greater than 125 nm. Degradation of the BET protein and downstream effects on Myc levels after treatment with macroPROTAC‐1 or MZ1 were next assessed in a disease‐relevant cell line, 22RV1 (human prostate carcinoma, Figure 4 A,B). MacroPROTAC‐1 induced the degradation of more than 90 % of Brd4 at 250 nm. MacroPROTAC‐1 also induced a more than 90 % depletion of cellular levels of Myc in 22RV1 cells, as downstream effect of BET degradation, with potency and kinetics comparable to MZ1 (Figure 4 A,B).

We next evaluated the impact of compound **1** on the viability of BET‐sensitive human prostate carcinoma 22RV1 and acute myeloid leukemia MV4;11 cancer cell lines (Figure 4 C,D). MacroPROTAC‐1 induced a marked antiproliferative effect with an EC_50_ of 640 nm in 22RV1 cells (Figure 4 C and Supporting Information) and an EC_50_ of 300 nm in MV4;11 cells (Figure 4 D and Supporting Information), a potency comparable to MZ1. Compound *cis*MZ1, which does not induce BET‐protein degradation but can still inhibit the BET proteins, was used as a reference control to confirm that cytotoxicity is due to BET‐protein degradation and not inhibition.^7^ Taken together, these results show that macroPROTAC‐1 has a very similar cellular activity to MZ1, despite its 12‐fold weaker binary binding affinity at the bromodomain end.

## Conclusion

In conclusion, this work illustrates a successful roadmap to macrocyclization PROTAC design strategies. Macrocyclization allows the constraint of a PROTAC molecule in its bioactive conformation, biasing it to adopt or discriminate against a desired ternary complex, which we show can aid degradation potency and selectivity amongst homologous targets. Our work also highlights that the binary binding energies of the PROTAC for the target protein (and E3 ligase) should ideally not be detrimentally impacted as a result of the cyclization process, and we suggest this should be monitored closely in future applications. We anticipate that structure‐based design of macrocyclic PROTACs will be an increasingly attractive and feasible strategy of drug design, in particular as relevant structural information continues to emerge and impact the field.^31^

## Conflict of interest

The Ciulli laboratory receives or has received sponsored research support from Boehringer Ingelheim, Eisai Inc., Nurix Inc., and Ono Pharma. A.C. is a scientific founder, director, and shareholder of Amphista Therapeutics, a company that is developing targeted protein degradation therapeutic platforms.

## Supporting information

As a service to our authors and readers, this journal provides supporting information supplied by the authors. Such materials are peer reviewed and may be re‐organized for online delivery, but are not copy‐edited or typeset. Technical support issues arising from supporting information (other than missing files) should be addressed to the authors.

Supplementary
