# Supplementary material for: Structure‐Based Design of a Macrocyclic PROTAC
Source: Angew Chem Int Ed Engl. 2019 Dec 12;59(4):1727–34. doi: 10.1002/anie.201914396 (PMC7004083; doi:10.1002/anie.201914396)
Supplement: Supplementary file 1 — Supplementary [file ANIE-59-1727-s001.pdf]

Angewandte Chemie
Eine Zeitschrift der Gesellschaft Deutscher Chemiker

Supporting Information

Structure-Based Design of a Macrocyclic PROTAC

*Andrea Testa, Scott J. Hughes, Xavier Lucas, Jane E. Wright, and Alessio Ciulli**

anie_201914396_sm_miscellaneous_information.pdf

Table of Contents

Supplementary Figures 2
Computational methods 9
Chemistry 11
Protein expression and purification 22
Isothermal titration calorimetry (ITC) 22
Fluorescence polarization assay 24
Crystallography 24
Tissue culture 27
Testing compounds in cells 27
Immunoblotting 27
Cell Viability Assay 28
Copies of NMR spectra 29

Supplementary Figures

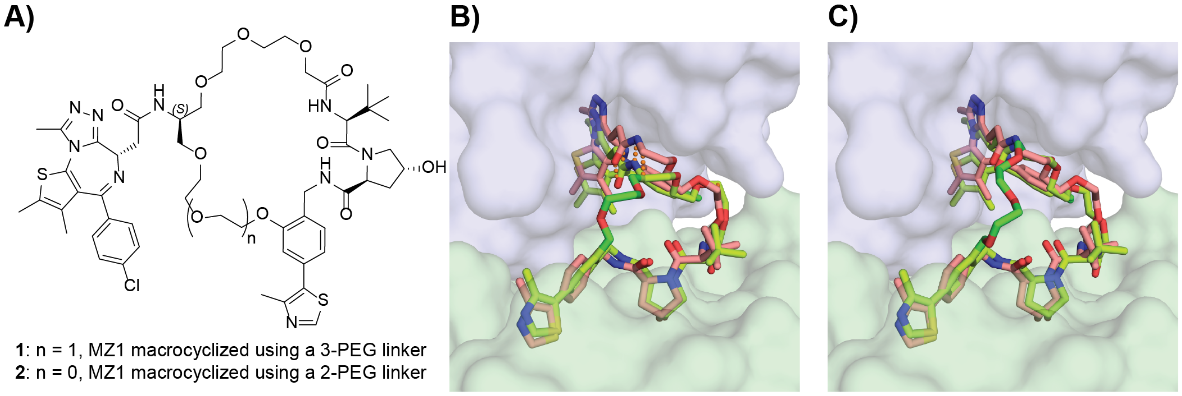

Figure S1. Structure and modeling of macrocyclic MZ1 derivatives. *(A) Chemical structures of cyclized derivatives* ***1*** *and* ***2****. (B, C) Superposition of the crystallographic pose of MZ1 (in salmon) and the energy-minimized structure of macrocyclic derivatives with a linker comprising (B) 2 PEG units and (C) 3 PEG units (in lime, with the new PEG linkers colored green). The surface of VHL and Brd4*^{BD2} *is colored pale green and light blue, respectively. In (B), atomic displacements > 1.5 Å upon energy-minimization are highlighted in dotted orange lines. No such large rearrangements were observed in (C), suggesting that the linker was more appropriate.*

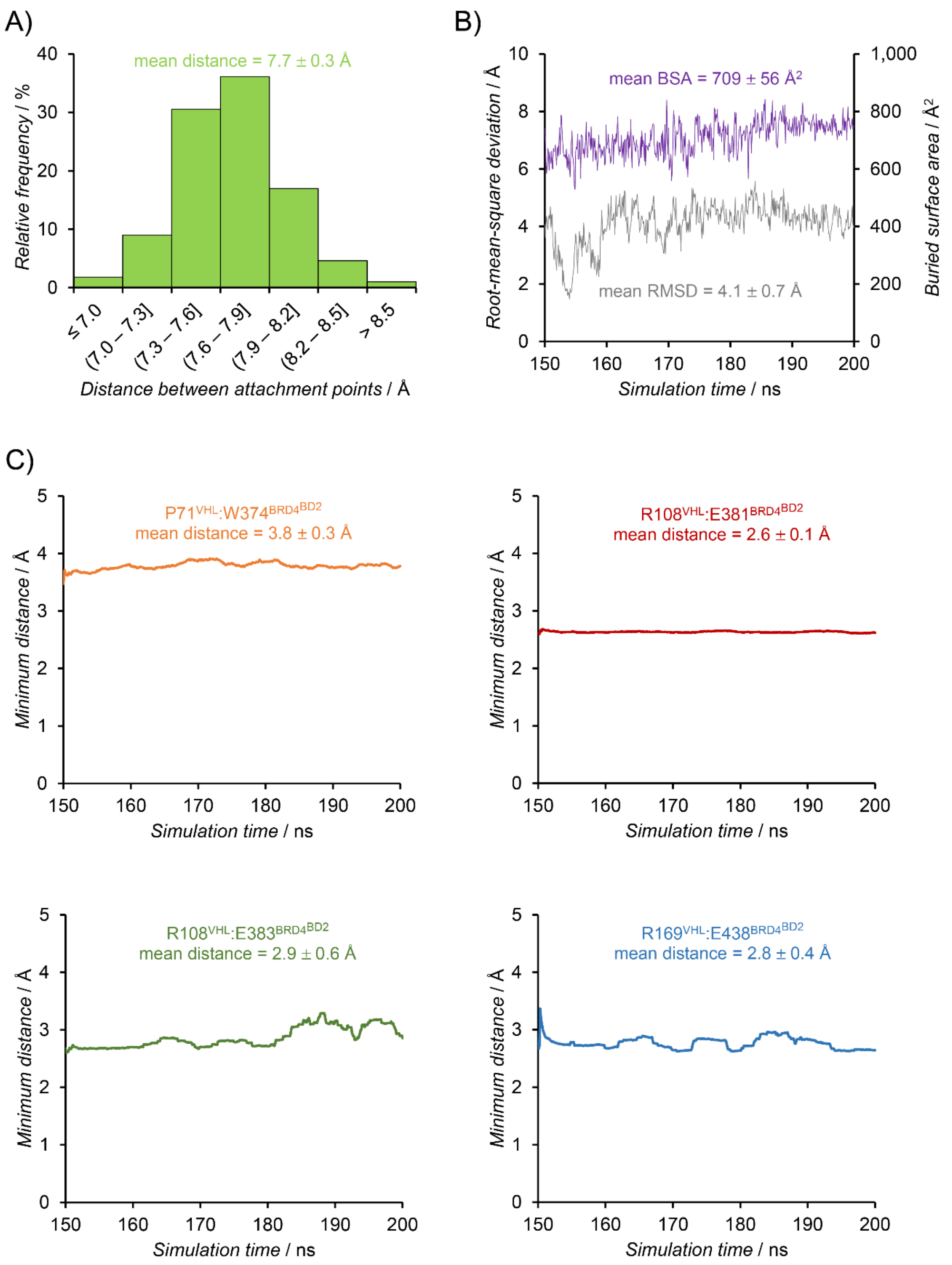

Figure S2. Structural integrity and stability of the VHL:1:Brd4^{BD2} ternary complex along the MD simulation. *(A) Histogram depicting the observed relative frequency of binned distances between the attachment points in compound* ***1****. (B) Buried surface area (BSA) in the interface of VHL with Brd4^{BD2} and root-mean-square deviation (RMSD) of all α-carbon atoms in the proteins, after superposition to the crystal structure of VCB:MZ1:Brd4^{BD2} (PDB code 5T35).[1] (C) Minimum distance between selected amino acids in VHL and Brd4^{BD2} that participate in protein–protein interactions.[1] For clarity, the plotted distances are the average over 5 ns. In all cases, only the last 50 ns in 100 ps intervals of a 200-ns MD simulation of the VHL:*__*1*__*:Brd4^{BD2} ternary complex were considered to generate the data. The mean ± 1 s.d. of each measurement is shown.*

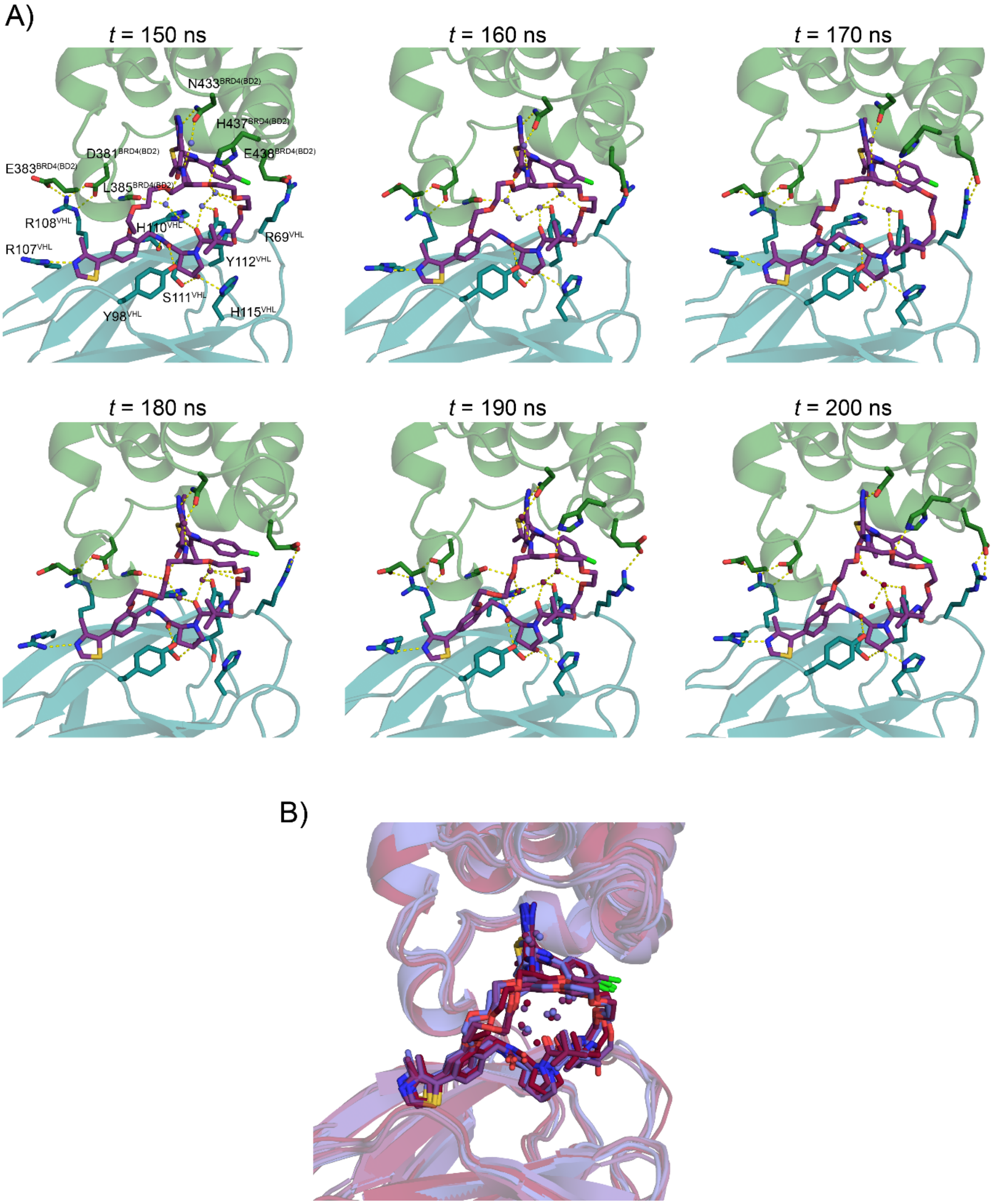

Figure S3. Interactions in the VHL:1:Brd4^{BD2} ternary complex along the MD simulation. *(A) Protein–protein and protein–ligand interactions (showed in dashed yellow lines) observed at 10 ns intervals along the last 50 ns of a 200-ns MD simulation of the VHL:**1**:Brd4^{BD2} ternary complex. A conserved water network stabilized at the interface of the three partners is shown along the simulation. In the first panel, interacting amino acids in VHL and Brd4^{BD2} are labelled. (B) Superposition of the frames showed in (A), with the time lapse colored from blue to dark red. Water molecules within the network form clusters at specific locations of the interface, indicating that they engage in stabilizing interactions with their surrounding during the simulation.*

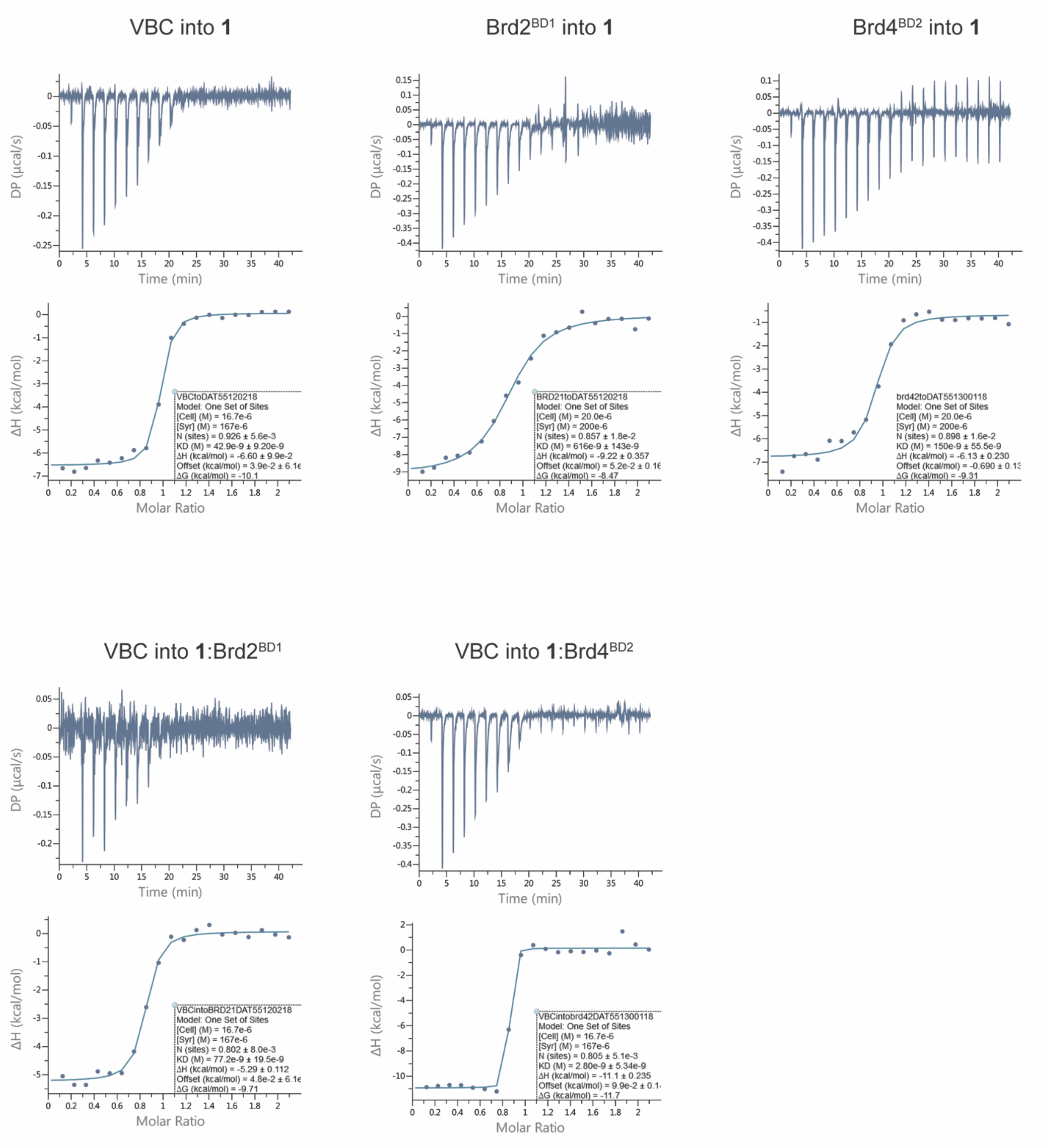

Figure S4. Representative ITC titrations obtained for compound 1 and VCB, Brd2^{BD1} and Brd4^{BD2}.

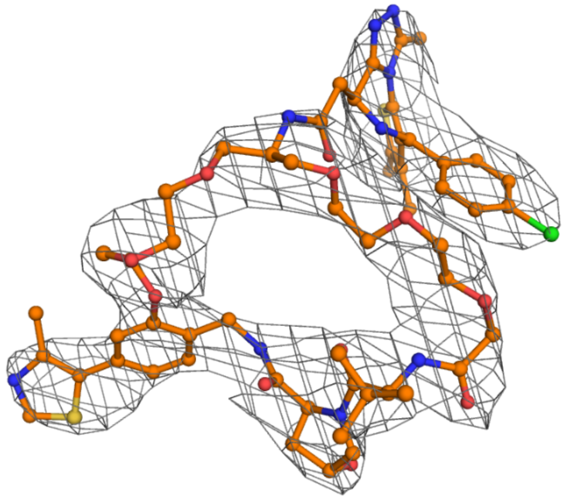

Figure S5. Electron density map of the ligand. *Fo – Fc omit map of compound **1** (contoured at 2.8 σ; carve = 2) showing electron density for the additional linker.*

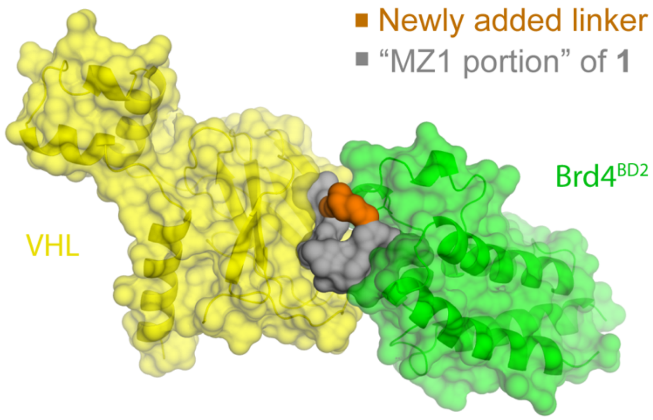

Figure S6. Surface representation of VCB:1:Brd4^{BD2} ternary complex. *Highlighted is the cavity filled by the "MZ1-portion"(gray) and additional cyclizing linker (orange) of compound **1**.*

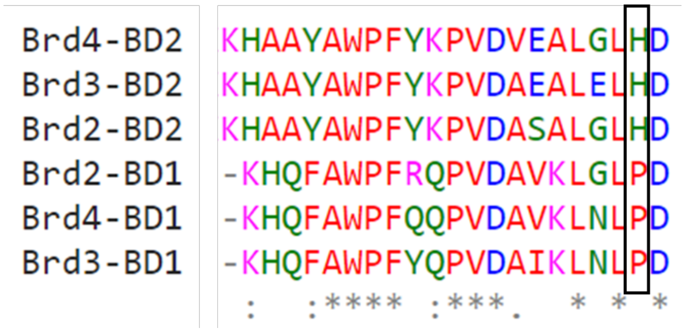

Figure S7. Sequence alignment of ZA-loop from the bromodomains of Brd2, Brd3 and Brd4. *Highlighted is the presence of a proline in the ZA-loop of BD1s, which is a histidine in the same position in the BD2s.*

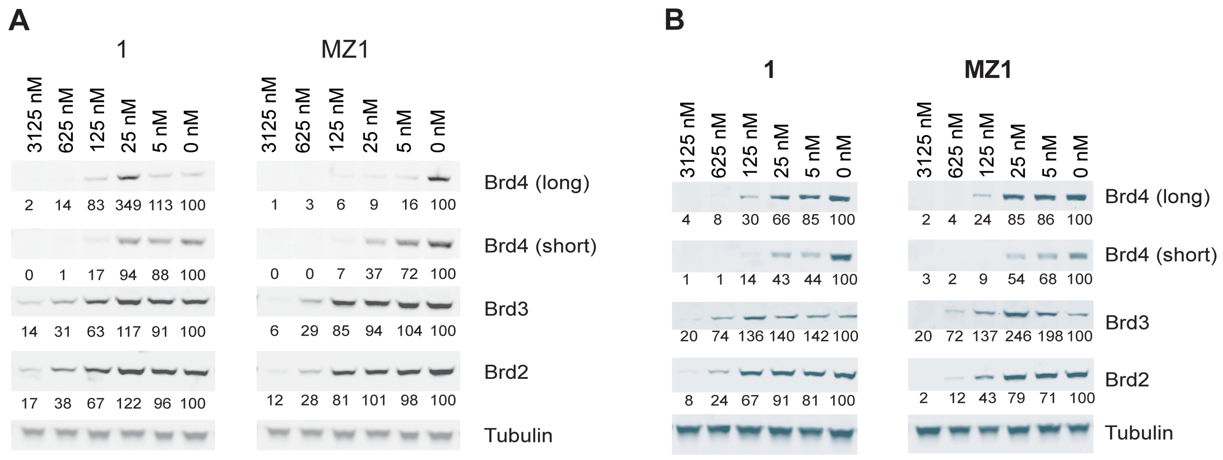

Figure S8. BET degradation profiles of compounds 1 and MZ1. *(A-B) Protein levels in HeLa cells after treatment with compound 1 or MZ1 for 18h (A) or 4h (B) visualised and quantified by Western blot.*

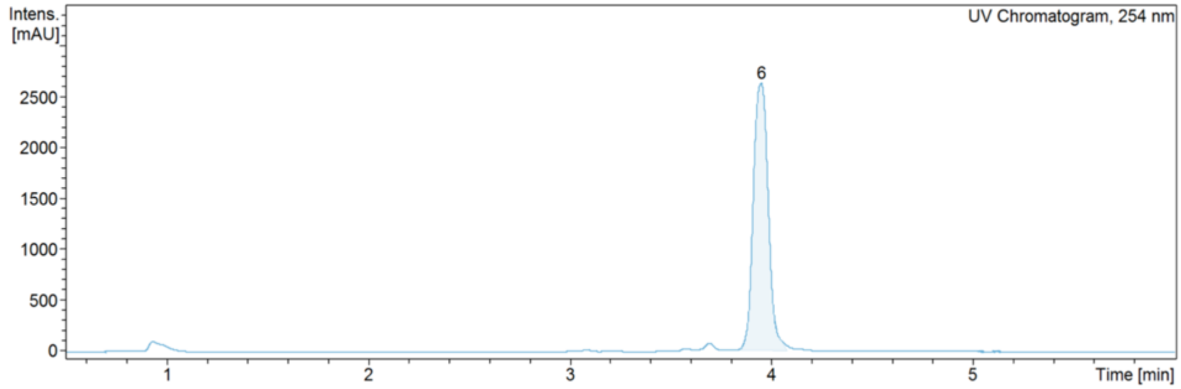

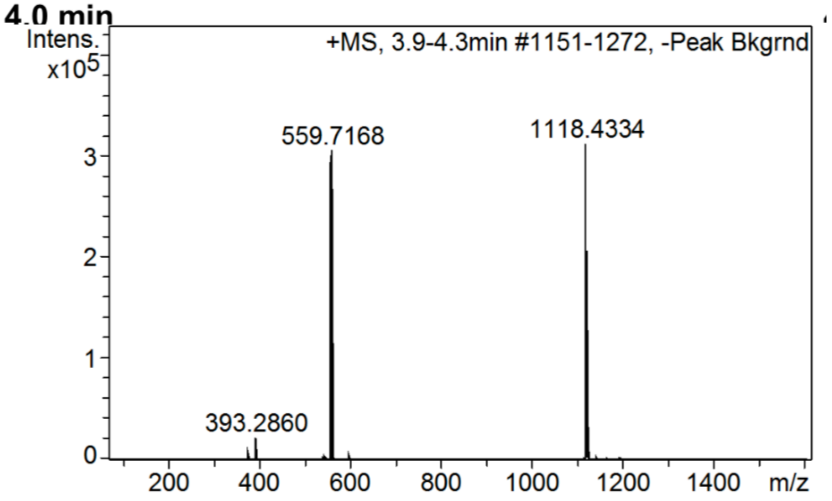

Figure S9. Analytical HPLC trace (254 nm) and HRMS of purified compound 1.

Computational methods

Torsion analysis of surrogate N-substituted acetamides

Model compounds *N*-ethylacetamide and *N*-isopropylacetamide (Fig. 1C) were subjected to a relaxed torsion scan analysis of their alkylacetamide bond using density functional theory (DFT) at the PBF (water) MN15-L/aug-cc-pVTZ(-F) level of theory in Jaguar 9.7 (Schrödinger, LLC). The *C–N–C–C* torsion was gradually rotated from 0 to 180º in 10º steps, considering Abelian molecular symmetry when appropriate.

Molecular modeling of macrocyclic MZ1 derivatives

Derivatives of MZ1 macrocyclized using a 2-PEG and a 3-PEG linker (Fig. 1D) were modeled *in situ* using the VHL:MZ1:Brd4^{BD2} ternary complex crystal structure as template and Glide Ligand Designer (Schrödinger). Water and solvent molecules present in the protein crystal structure were removed, and the VHL:MZ1:Brd4^{BD2} system was prepared for energy minimization in Prime (Schrödinger) using the Protein Preparation Wizard (Schrödinger). Amino acid protonation states were assigned using PROPKA 3.0.[2]

*Molecular dynamics (MD) simulation of the VHL:**1**:Brd4^{BD2} ternary complex*

The energy-minimized model of VHL:**1**:Brd4^{BD2} was used as starting point for a molecular dynamics (MD) simulation. MD simulations were carried out in an nVIDIA TITAN X GPU using Desmond (Schrödinger) and the OPLS3 force field.[3] System Builder (Schrödinger) was used to solvate the complex in a TIP3P water box with a padding of 10 Å from the edge of the box to any solute atom, and to neutralize the system charges with three chloride ions. The solvated system was minimized for 2,000 steps with all protein and PROTAC atoms restrained to eliminate residual unfavorable interactions between the solute and the solvent, followed by another 2,000 steps with unrestrained PROTAC and solvent atoms, and lastly followed by another 5,000 steps with all atoms free to move. The equilibration phase consisted of an initial 100-ps Brownian dynamics simulation at 10 K with restraints on solute heavy atoms in the NVT ensemble, followed by one run of 120 ps of MD at the same temperature, restraints, and ensemble. The restrained system was

then subjected to two runs of 120 ps in the NPT ensemble, first at 10 K and then at 300 K, and a final unrestrained simulation at the final temperature (300 K) for 240 ps. We run 200 ns of MD (time step of 2 fs using the RESPA integrator), starting from the equilibrated system. The temperature was controlled with a Nose-Hoover chain thermostat at 300 K, and the pressure with a Martyna-Tobias-Klein barostat at 1 bar. Short-range nonbonded interactions were cut off at 9 Å. Only the last 50 ns of production simulation were used for data collection and analysis.

Analysis of MD trajectories

The last 50 ns of the 200-ns MD trajectory were analyzed using the Simulation Quality, Event, and Interactions Analysis tools, as included in Schrödinger. The buried surface area (BSA) of the proteins upon complex formation, *i.e.* the difference in surface-accessible surface area (SASA) between the formed complex and the unbound partners in each system, was computed using VMD v. 1.9.2 [4] and considering all protein atoms and a spherical probe of radius 1.4 Å.

System	*Number of atoms*	*Total energy* / Mcal mol^{-1}	*Pressure* / bar	*Volume* / nm^3	*Temperature* / K
VHL:1:Brd4^{BD2}	131,674	–335.6 ± 0.1	1 ± 65	1,328 ± 2	298.7 ± 0.8

Table S 1. Stability of the MD simulation of VHL:1:Brd4^{BD2}. *Number of atoms and physical properties of the system under simulation during the last 50 ns of a 200-ns MD simulation. Values given are the mean ± 1 s.d.*

Chemistry

All chemicals, unless otherwise stated were commercially available and used without further purification. Solvents were anhydrous and reactions preformed under positive pressure of nitrogen or argon. Enantiopure (+)-JQ-1 was purchased from Medchemexpress LLC, Princeton, USA. Flash column chromatography (FCC) was performed using a Teledyne Isco Combiflash Rf or Rf200i. As prepacked columns RediSep Rf Normal Phase Disposable Columns were used.

NMR spectra were recorded on a Bruker 500 Ultrashield or a Bruker Ascend 400. Chemical shifts are quoted in ppm and referenced to the residual solvent signals: ^{1}H δ = 7.26 ($CDCl_3$), ^{13}C δ = 77.0 ($CDCl_3$), ^{1}H δ = 3.32 (MeOD), ^{13}C δ = 49.15 (MeOD), ^{1}H δ = 2.50 (DMSO-D6), ^{13}C δ = 39.51(DMSO-D6). Signal splitting patterns are described as singlet (s), doublet (d), triplet (t), quartet (q), multiplet (m), broad (br) or a combination thereof. Coupling constants (*J*) are measured in Hz. Low resolution MS and analytical HPLC traces were recorded on an Agilent Technologies 1200 series HPLC connected to an Agilent Technologies 6130 quadrupole LC/MS, connected to an Agilent diode array detector. The column used was a Waters XBridge column (50 mm × 2.1 mm, 3.5 µm particle size) and the compounds were eluted with a gradient of 5−95% acetonitrile/water + 0.1% formic acid over 3 min (METHOD 1) or over 7 min (METHOD 2). Preparative HPLC was performed on a Gilson Preparative HPLC System with a Waters X-Bridge C18 column (100 mm × 19 mm; 5 µm particle size).

Abbreviations used: ACN for acetonitrile, COMU for (1-cyano-2-ethoxy-2-oxoethylidenaminooxy)dimethylamino-morpholino-carbenium hexafluorophosphate, DCM for dichloromethane, EtOAc for ethyl acetate, DMSO for dimethyl sulfoxide, DIPEA for N,N-diisopropylethylamine, FCC for flash column chromatography, MeOH for methanol, TEA for triethylamine, DMF for N,N-dimethylformamide, HATU for 1-[bis(dimethylamino)methylene]-1H-1,2,3-triazolo[4,5-b]pyridinium 3-oxid hexafluorophosphate, TFA for trifluoroacetic acid.

(S)-4-((2-(2-(benzyloxy)ethoxy)ethoxy)methyl)-2,2-dimethyl-1,3-dioxolane (5)

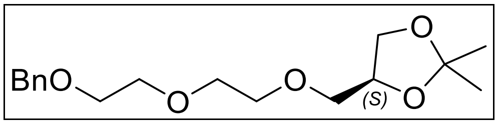

To a solution of (*S*)-(+)-1,2-isopropylideneglycerol **3** (1.6 g, 12.0 mmol) in dioxane (40 mL) freshly ground KOH (740 mg, 13.2 mmol) was added, followed by 2-(2-(benzyloxy)ethoxy)ethyl 4-methylbenzenesulfonate **4** (7.0 g, 20 mmol) and a catalytic amount of tetrabutylammonium iodide. The mixture was stirred at 105 °C overnight. The solvent was removed under reduced pressure, a saturated solution of ammonium chloride (50 mL) was added to the residue and the mixture was extracted with DCM (3 × 100 mL). The collected organic phase was dried on anhydrous $MgSO_4$, evaporated to dryness and the crude was purified by FCC using a gradient from 10% to 50% of EtOAc in heptane. Obtained 1.94 g, 52% (transparent oil).

^{1}H-NMR (500 MHz, $CDCl_3$) δ: 7.35 - 7.25 (m, 5H), 4.27 (td, *J*=6.0, 12.0 Hz, 1H), 4.04 (dd, *J*=6.4, 8.2 Hz, 1H), 3.74 - 3.61 (m, 9H), 3.58 (dd, *J*=5.9, 9.8 Hz, 1H), 3.50 (dd, *J*=5.7, 10.0 Hz, 1H), 1.41 (s, 3H), 1.35 (s, 3H).

^{13}C-NMR (12.5.77 MHz, $CDCl_3$) δ: 138.2, 128.3, 127.7, 127.6, 109.3, 74.7, 73.2, 72.4, 71.0, 70.7, 70.6, 69.4, 66.8, 26.8, 25.4.

(R)-3-(2-(2-(benzyloxy)ethoxy)ethoxy)propane-1,2-diol (6)

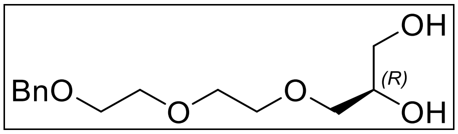

The acetonide **5** (1.94 g, 6.25 mmol) was dissolved in 80% aqueous acetic acid (40 mL) and stirred until complete consumption of the starting material was observed by TLC (50% of EtOAc in heptane). The reaction mixture was evaporated to dryness to obtain the desired product in quantitative yield (1.68 g) as a transparent oil.

^{1}H-NMR (500 MHz, $CDCl_3$) δ: 7.35 - 7.27 (m, 5H), 4.57 (s, 2H), 3.88 - 3.83 (m, 1H), 3.70 - 3.54 (m, 12H), 2.08 - 2.07 (m, 1H).

^{13}C-NMR (125.77 MHz, $CDCl_3$) δ: 138.0, 128.4, 127.8, 127.7, 73.3, 73.0, 70.8, 70.6, 70.5, 69.4, 63.9.

(S)-13,13,14,14-tetramethyl-1-phenyl-2,5,8,12-tetraoxa-13-silapentadecan-10-ol (7)

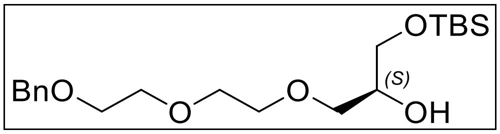

A solution of **6** (1.68 g, 6.25 mmol) in anhydrous DCM (7 mL) was cooled at 0 °C. TEA (1.2 mL, 8.75 mmol) and DMAP (46 mg, 0.375 mmol) were added, followed by a dropwise addition of TBSCl (1.32 g, 8.75 mmol) in DCM (8 mL). The reaction was allowed to reach r.t. overnight while stirring, then the reaction mixture was quenched with water (20 mL) and extracted with DCM. The organic layer was dried over $MgSO_4$ and evaporated to dryness. The crude product was purified by FCC using a gradient from 20% to 50% of EtOAc in heptane. Obtained 2.0 g, 83% yield (transparent oil).
^{1}H-NMR (500 MHz, $CDCl_3$) δ: 7.35 - 7.27 (m, 5H), 4.57 (s, 2H), 3.85 - 3.79 (m, 1H), 3.69 - 3.62 (m, 10H), 3.57 (dd, *J*=4.7, 10.0 Hz, 1H), 3.50 (q, *J*=5.3 Hz, 1H), 2.65 (d, *J*=4.8 Hz, 1H), 0.89 (s, 9H), 0.06 (s, 6H).
^{13}C-NMR (125.77 MHz, $CDCl_3$) δ: 138.2, 128.4, 127.8, 127.6, 73.3, 72.3, 70.9, 70.7, 70.6, 69.4, 64.0, 25.9, 18.3, -5.4.

(R)-10-azido-13,13,14,14-tetramethyl-1-phenyl-2,5,8,12-tetraoxa-13-silapentadecane (8)

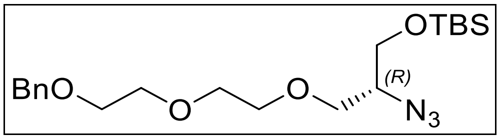

To a solution of **7** (2.0 g, 5.2 mmol) in anhydrous pyridine (11 mL), mesyl chloride (0.800 mL, 10.4 mmol) was added dropwise. The was allowed to reach r.t. overnight while stirring, then the reaction mixture was quenched with saturated sodium bicarbonate (20 mL) and extracted with DCM. The organic layer was dried over $MgSO_4$ and concentrated before being dissolved in DMF (20 mL). Sodium azide (1.39 g, 26 mmol) was added and the reaction mixture was heated at 100

°C overnight. The reaction mixture was then allowed to cool to r.t., saturated sodium bicarbonate (20 mL) was added the mixture was extracted with EtOAc. The organic layer was dried over $MgSO_4$ and concentrated. The crude was then purified by FCC using a gradient from 0% to 50% of EtOAc in heptane. Obtained 0.95 g, 45% yield over two steps (transparent oil).

^{1}H-NMR (400 MHz, $CDCl_3$) δ: 7.35 - 7.26 (m, 5H), 4.57 (s, 2H), 3.77 - 3.52 (m, 13H), 0.90 (s, 9H), 0.08 (s, 6H).

^{13}C-NMR (100.62 MHz, $CDCl_3$) δ: 138.2, 128.3, 127.7, 127.6, 73.2, 70.9, 70.7, 70.6, 70.5, 69.4, 63.3, 62.3, 25.8, 18.2, -5.6.

(R)-10-azido-1,1,1,19-tetraphenyl-2,5,8,12,15,18-hexaoxanonadecane (10)

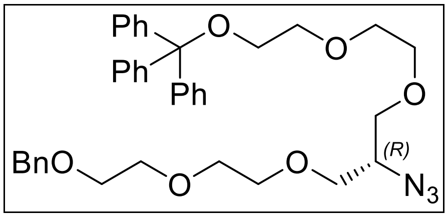

To a solution of **8** (952 mg, 2.06 mmol) in anhydrous THF (10 mL) a solution of TBAF (1 M in THF, 6.2 mL) was added dropwise at 0 °C. The ice bath was removed and the reaction mixture was allowed to stir at r.t. until complete consumption of the starting material was observed by TLC (40% of EtOAc in heptane). A saturated solution of ammonium chloride (10 mL) was added the mixture was extracted with DCM. The organic layer was dried over $MgSO_4$ and concentrated. ^{1}H-NMR (500 MHz, $CDCl_3$) d: 7.35 - 7.27 (m, 5H), 4.57 (s, 2H), 3.78 - 3.62 (m, 14H), 2.29 (t, J=6.2 Hz, 1H)].

The deprotected alcohol (620 mg, 2.0 mmol) was then dissolved in dioxane (20 mL), freshly ground KOH (168 mg, 3 mmol) was added, followed by 2-(2-(trityloxy)ethoxy)ethyl methanesulfonate [5] (**9**, 980 mg, 2.3 mmol) and TBAI (85 mg, 0.23 mmol). The mixture was stirred at 105 °C overnight. ~The mixture was allowed to cool to r.t., then brine (20 mL) was added and the mixture was extracted with DCM (3 × 100 mL). The collected organic phase was dried on anhydrous $MgSO_4$, evaporated to dryness and the crude was purified by FCC using a gradient from 10% to 70% of EtOAc in heptane. Obtained 1.32 g, 97% yield (transparent oil).

^{1}H-NMR (400 MHz, $CDCl_3$) δ: 7.41 - 7.36 (m, 7H), 7.27 - 7.12 (m, 16H), 4.49 (s, 1H), 3.67 - 3.44 (m, 20H), 3.17 (t, *J*=4.8 Hz, 2H).
^{13}C-NMR (100.62 MHz, $CDCl_3$) δ: 144.2, 138.3, 128.7, 128.4, 127.8, 127.7, 127.6, 126.9, 86.6, 73.3, 71.1, 71.0, 70.8, 70.6, 69.5, 63.4, 60.7.

(S)-10-azido-1-phenyl-2,5,8,12,15-pentaoxaheptadecan-17-ol (11)

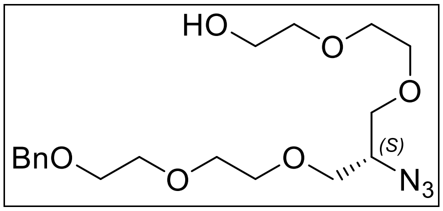

To a solution of **10** (600 mg, 0.95 mmol) in DCM (9 ml) TFA (1 mL) was added, followed by TIPS (1mL). The reaction mixture was stirred for 20 min, TLC (40% of EtOAc in heptane) analysis showed complete consumption of the starting material. The reaction was quenched with a saturated solution of $NaHCO_3$ and extracted with DCM. The collected organic phase was dried on anhydrous $MgSO_4$, evaporated to dryness and the crude was purified by FCC using a gradient from 20% to 100% of EtOAc in heptane. Elution of the desired product was obtained with 100% EtOAc. Obtained 280 mg, 77% yield (transparent oil).
^{1}H-NMR (500 MHz, $CDCl_3$) δ: 7.35 - 7.28 (m, 5H), 4.58 (s, 2H), 3.80 - 3.55 (m, 22H).
^{13}C-NMR (100.62 MHz, $CDCl_3$) δ: 138.3, 128.4, 127.7, 127.6, 73.3, 72.4, 71.0, 70.9, 70.8, 70.7, 70.6, 70.4, 69.5, 61.8, 60.6.

tert-butyl (R)-10-azido-1-phenyl-2,5,8,12,15,18-hexaoxaicosan-20-oate (12)

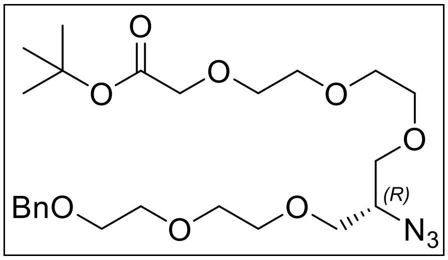

To a stirred solution of **11** (271 mg, 0.70 mmol) in DCM (1.5 mL) a solution of 37% NaOH (1.5 ml) was added, followed by *tert*-butylbromo acetate (546 mg, 2.8 mmol) and TBABr (230 mg, 0.71 mmol). The resulting solution was stirred overnight at r.t. The reaction mixture was diluted with water and extracted with DCM. The organic phase was over $MgSO_4$ and concentrate in vacuo. The resulting oil was purified by FCC using a gradient from 20% to 100% of EtOAc in heptane. Yield: 246 mg, 71%, transparent oil.

^{1}H-NMR (500 MHz, $CDCl_3$) δ: 7.35 - 7.27 (m, 5H), 4.57 (s, 2H), 4.01 (s, 2H), 3.74 - 3.53 (m, 21H), 1.48 (s, 9H).

^{13}C-NMR (125.77 MHz, $CDCl_3$) δ: 169.6, 138.3, 128.3, 127.7, 127.6, 81.5, 73.2, 71.0, 70.9, 70.7, 70.6, 70.5, 69.5, 69.0, 60.6, 28.1.

tert-butyl (R)-11-((2-(2-hydroxyethoxy)ethoxy)methyl)-13-oxo-3,6,9,14-tetraoxa-12-azaheptadec-16-enoate (13)

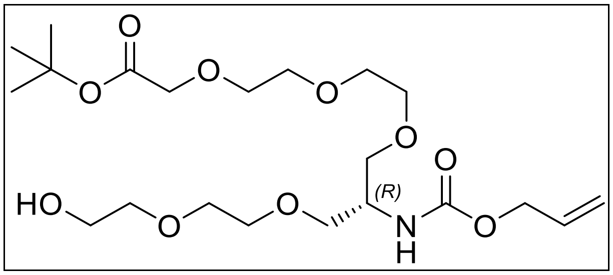

A solution of **12** (240 mg, 0.48 mmol) in ethanol (0.05 M) containing 5% of acetic acid was hydrogenated at 90 bar in a H-Cube (ThalesNano, 10% Pd/C cartridge) using a flow of 0.25 mL/min, 70 °C. The solvent was removed under reduced pressure and the crude was dissolved in dioxane/water (2:1, 3 mL). $NaHCO_3$ (130 mg, 1.53mmol) and *N*-(allyloxycarbonyloxy)succinimide (123 mg, 0.614 mmol) were added and the reaction was stirred at room temperature overnight .The reaction mixture was extracted with DCM, the organic phase was over $MgSO_4$ and concentrate in vacuo. The crude was purified by by FCC using a gradient from 0% to 10% of MeOH in DCM. Yield: 197 mg, 88% over two steps, transparent glass.

^{1}H-NMR (500 MHz, $CDCl_3$) δ: 5.96 - 5.86 (m, 1H), 5.47 (d, *J*=7.5 Hz, 1H), 5.32 - 5.19 (m, 3H), 4.58 - 4.52 (m, 2H), 4.01 (s, 2H), 3.74 - 3.49 (m, 21H), 1.45 (s, 9H).

^{13}C-NMR (100.62 MHz, $CDCl_3$) δ: 169.6, 155.9, 132.9, 117.7, 81.5, 72.5, 70.7, 70.6, 70.5, 70.4, 69.7, 69.6, 69.0, 65.7, 65.5, 61.8, 50.2, 28.1. MS analysis: m/z = 466.3 $[M+H]^+$, expected for $C_{21}H_{39}NO_{10}$: 465.3.

tert-butyl (S)-11-((2-(2-(2-(((2S,4R)-1-((S)-2-((tert-butoxycarbonyl)amino)-3,3-dimethylbutanoyl)-4-hydroxypyrrolidine-2-carboxamido)methyl)-5-(4-methylthiazol-5-yl)phenoxy)ethoxy)ethoxy)methyl)-13-oxo-3,6,9,14-tetraoxa-12-azaheptadec-16-enoate (16)

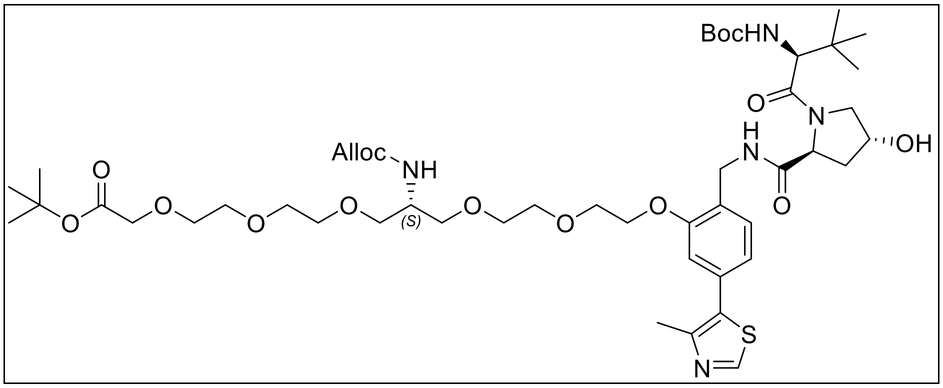

To a solution of **13** (187 mg, 0.40 mmol) in DCM (3 mL) at 0 °C, TEA (84 µL, 0.60 mmol) was added, followed by mesyl chloride (37 µL, 0.48 mmol). The reaction mixture was stirred at 0 °C for 10 min and then allowed to reach r.t. over 3 h. A 5% solution of $NaHCO_3$ was added, the reaction mixture was extracted with DCM, the organic phase was over $MgSO_4$ and concentrate in vacuo to give the mesylate linker which was immediately dissolved in anhydrous DMF (2 mL). Compound **15** [6] (145 mg, 0.27 mmol) was added to the mesylate solution, followed by freshly ground anhydrous K_2CO_3 (110 mg, 0.80 mmol). The reaction mixture was vigorously stirred at 75 °C overnight. The reaction mixture was then allowed to cool to r.t., diluted with DCM and filtered over a celite pad. Volatiles were removed under reduced pressure and the crude was purified by FCC using a gradient from 0% to 15% of MeOH in DCM. Yield: 169 mg, 64% over two steps, transparent glass.

^{1}H-NMR (400 MHz, MeOD) δ: 8.89 (s, 1H), 7.48 (d, *J*=7.8 Hz, 1H), 6.37 (d, *J*=9.5 Hz, 1H), 5.97 - 5.87 (m, 1H), 5.34 - 5.15 (m, 3H), 4.63 (t, *J*=8.3 Hz, 1H), 4.57 - 4.37 (m, 7H), 4.32 - 4.23 (m, 4H), 4.02 (s, 2H), 3.94 - 3.86 (m, 5H), 3.82 - 3.74 (m, 4H), 3.69 - 3.53 (m, 21H), 2.50 (s, 3H), 2.26 - 2.20 (m, 1H), 2.15-2.05 (m, 1H), 1.48 (s, 9), 1.45 (s, 9H), 1.00 (s, 9H).

^{13}C-NMR (100.62 MHz, MeOD) δ: 173.1, 171.9, 158.3, 153.2, 149.4, 134.8, 133.8, 133.1, 130.3, 128.7, 123.2, 117.9, 114.1, 83.0, 81.0, 72.0, 72.0, 71.8, 71.8, 71.5, 71.4, 71.2, 70.1, 69.7, 66.7, 61.0, 60.6, 58.3, 52.4, 39.8, 39.1, 37.1, 29.0, 28.7, 27.2, 16.2. MS analysis: m/z = 994.5 $[M+H]^+$, expected for $C_{48}H_{75}N_5O_{15}S$: 993.5.

allyl ((2R,6S,18S,33aS)-6-(tert-butyl)-2-hydroxy-28-(4-methylthiazol-5-yl)-5,8,33-trioxo-2,3,6,7,8,9,11,12,14,15,18,19,21,22,24,25,31,32,33,33a-icosahydro-1H,5H,17H-benzo[r]pyrrolo[1,2-m][1,4,7,20,23,26]hexaoxa[10,13,16]triazacyclononacosin-18-yl)carbamate (17)

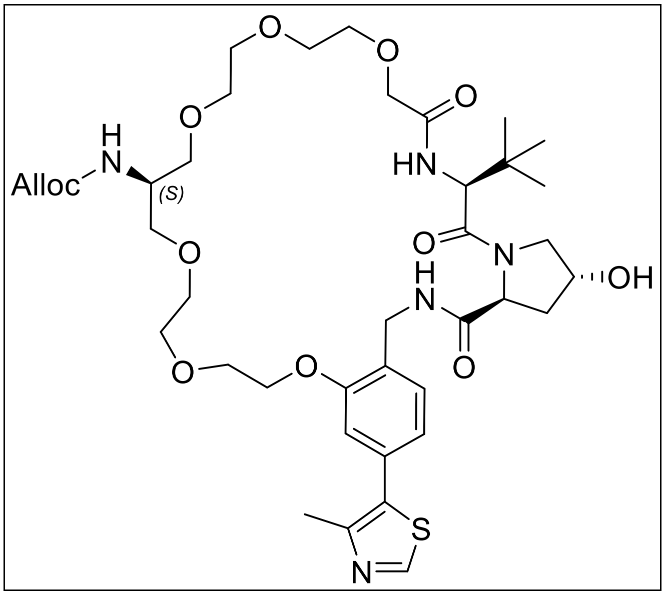

Compound **16** (169 mg, 0.17 mmol) was dissolved in a mixture of TFA/DCM (1:1, 3 mL). The reaction was stirred for 3 h, then volatile components were evaporated under reduced pressure. The reside was taken up with 0.1 M aqueous HCl (15 mL), frozen and freeze dried to obtain the HCl salt which was then dissolved in a mixture of anhydrous DCM (50 mL) and DIPEA (110 µL, 0.63 mmol). This solution was added dropwise to a solution of HATU (162 mg, 0.425 mmol) and DIPEA (55 µL, 0.32 mmol) in DCM (50 mL). The reaction mixture was stirred overnight. Solvent was removed and the crude was purified by FCC using a gradient from 0% to 20% of MeOH in DCM. Yield: 105 mg, 75% over two steps, transparent glass.

^{1}H-NMR (400 MHz, MeOD) δ: 8.89 (s, 1H), 8.22 (t, *J*=5.7 Hz, 1H), 7.67 (d, *J*=10.3 Hz, 1H), 7.36 (d, *J*=8.0 Hz, 1H), 7.08 (d, *J*=1.3 Hz, 1H), 7.03 (d, *J*=1.7 Hz, 1H), 7.02 (d, *J*=1.4 Hz, 1H), 5.97 -

5.87 (m, 1H), 5.29 (dd, *J*=1.5, 17.2 Hz, 1H), 5.15 (d, *J*=10.6 Hz, 1H), 4.74 (d, *J*=9.7 Hz, 1H), 4.63 - 4.44 (m, 5H), 4.39 - 4.28 (m, 2H), 4.19 - 4.12 (m, 1H), 4.02 (s, 2H), 3.98 - 3.53 (m, 23H), 2.52 (s, 3H), 2.23 - 2.16 (m, 1H), 1.97 - 1.90 (m, 1H), 1.05 (s, 9H).

^{13}C-NMR (100.62 MHz, MeOD) δ: 174.3, 172.2, 172.1, 171.4, 158.8, 153.2, 149.5, 134.7, 133.6, 131.4, 129.3, 123.3, 117.9, 115.0, 72.7, 72.3, 71.9, 71.8, 71.6, 71.4, 71.2, 70.7, 69.8, 60.8, 58.3, 52.6, 40.4, 39.1, 37.8, 27.3, 16.2. MS analysis: m/z = 820.4 $[M+H]^+$, expected for $C_{39}H_{57}N_5O_{12}S$: 819.4.

(2R,6S,18S,33aS)-18-amino-6-(tert-butyl)-2-hydroxy-28-(4-methylthiazol-5-yl)-1,2,3,6,7,11,12,14,15,18,19,21,22,24,25,31,32,33a-octadecahydro-5H,17H,33H-benzo[r]pyrrolo[1,2-m][1,4,7,20,23,26]hexaoxa[10,13,16]triazacyclononacosine-5,8,33(9H)-trione (18)

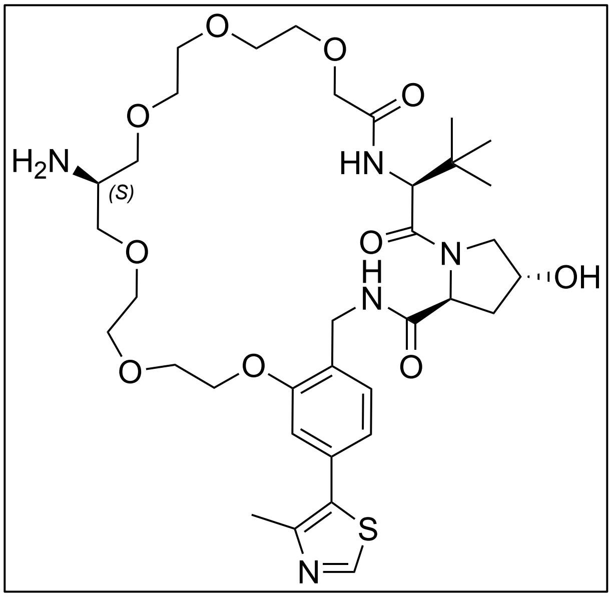

To a solution of **17** (35mg, 0.043 mmol) in THF (3 mL), $Pd(PPh_3)_4$ (5 mg, 0.0043 mmol) and phenylsilane (53 µL, 0.43 mmol) were added. The reaction was stirred for 20 min at r.t., dried over reduced pressure and the reside was dissolved in methanol, filtered and purified by preparative HPLC using a gradient from 5% to 95% of acetonitrile in water containing 0.01% of ammonium hydroxide over 15 min. The fractions congaing the product were freeze dried to obtain the desired product, 19 mg (63 %) as a white solid.

^{1}H-NMR (400 MHz, DMSO-D6) δ: 8.97 (s, 1H), 8.45 - 8.40 (m, 1H), 7.38 (d, *J*=9.4 Hz, 1H), 7.20 (d, *J*=7.6 Hz, 1H), 7.05 (d, *J*=1.5 Hz, 1H), 7.01 (dd, *J*=1.4, 7.8 Hz, 1H), 4.60 - 4.43 (m, 3H), 4.35 (s, 1H), 4.26 - 4.07 (m, 4H), 3.93 (s, 2H), 3.78 (t, *J*=5.3 Hz, 2H), 3.64 - 3.25 (m, 24H), 3.07 - 3.00 (m, 1H), 2.47 (s, 3H), 2.14 - 2.07 (m, 1H), 1.96 - 1.87 (m, 1H), 0.96 (s, 9H).
^{13}C-NMR (100.62 MHz, DMSO-D6) δ: 172.1, 169.4, 168.9, 165.0, 156.4, 151.9, 148.4, 131.7, 131.4, 128.0, 121.7, 113.1, 72.9, 72.7, 71.5, 70.6, 70.5, 70.4, 70.1, 70.0, 69.4, 69.3, 68.6, 59.3, 57.2, 56.2, 50.7, 38.3, 37.4, 36.5, 26.7, 16.5. MS analysis: m/z = 736.4 $[M+H]^+$, expected for $C_{35}H_{53}N_5O_{10}S$: 735.4.

N-((2R,6S,18S,33aS)-6-(tert-butyl)-2-hydroxy-28-(4-methylthiazol-5-yl)-5,8,33-trioxo-2,3,6,7,8,9,11,12,14,15,18,19,21,22,24,25,31,32,33,33a-icosahydro-1H,5H,17H-benzo[r]pyrrolo[1,2-m][1,4,7,20,23,26]hexaoxa[10,13,16]triazacyclononacosin-18-yl)-2-((S)-4-(4-chlorophenyl)-2,3,9-trimethyl-6H-thieno[3,2-f][1,2,4]triazolo[4,3-a][1,4]diazepin-6-yl)acetamide (1)

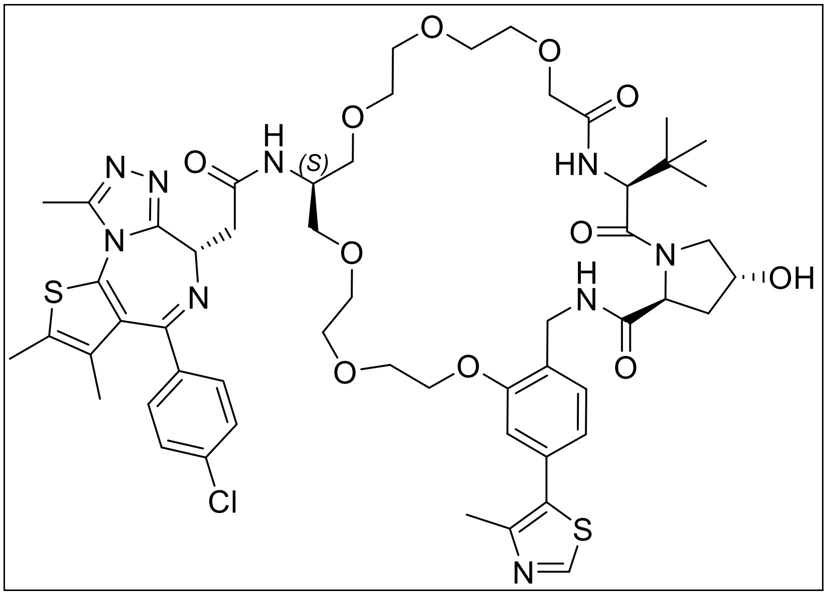

To a solution of amine **17** (10 mg, 0.136 mmol), (+)-JQ1-COOH [7] (5.5 mg, 0.0136 mmol) in DMF (1 mL), DIPEA (5 μL, 0.0272 mmol) and COMU (6 mg, 0.0136 mmol) were added. The reaction was stirred at r.t. for 1 h. Methanol (0.5 mL) was added and the crude mixture was purified

by preparative HPLC using a gradient from 5% to 95% of acetonitrile in water containing 0.01% of ammonium hydroxide over 15 min. The fractions congaing the product were freeze dried to obtain the desired product, 8 mg (52%) as a white solid.

^{1}H-NMR (400 MHz, MeOD) δ: 8.87 (s, 1H), 7.47 (d, *J*=8.5 Hz, 2H), 7.41 (d, *J*=8.5 Hz, 2H), 7.33 (d, *J*=7.9 Hz, 1H), 7.06 (d, *J*=1.4 Hz, 1H), 6.97 (dd, *J*=1.5, 7.8 Hz, 1H), 4.71 (s, 1H), 4.63 - 4.56 (m, 3H), 4.45 - 4.42 (m, 1H), 4.39 - 4.31 (m, 2H), 4.28 (t, J=5.6 Hz, 1H), 4.21 - 4.16 (m, 1H), 4.03 (s, 2H), 3.99 - 3.87 (m, 2H), 3.81 - 3.58 (m, 18H), 3.47 - 3.41 (m, 1H), 2.67 (s, 3H), 2.48 (s, 3H), 2.45 (s, 3H), 2.20 - 2.14 (m, 1H), 2.01 - 1.94 (m, 1H), 1.71 (s, 3H), 1.04 (s, 9H).

^{13}C-NMR (100.62 MHz, MeOD) δ: 174.4, 173.0, 172.1, 171.4, 166.3, 158.7, 157.3, 153.2, 152.4, 149.5, 138.4, 138.2, 133.4, 132.4, 131.7, 131.0, 130.1, 129.0, 123.1, 114.6, 72.7, 72.2, 72.1, 71.9, 71.4, 71.3, 71.2, 70.9, 69.8, 60.9, 58.3, 55.5, 50.9, 40.2, 39.1, 37.8, 27.4, 16.3, 14.7, 13.2, 11.9.

HRMS analysis: m/z = N1118.4334 $[M+H]^{+}$, 559.7168 $[M+2H]^{2+}$ expected for $C_{54}H_{68}ClN_{9}O_{11}S_{2}$: 1117.4168.

Protein expression and purification

Wild-type versions of human proteins VHL (UniProt accession number: P40337), ElonginC (Q15369), ElonginB (Q15370), Brd2 (P25440), Brd3 (Q15059) and Brd4 (O60885) were used for all protein expression. For expression of VCB, N-terminally His_6-tagged VHL (54–213), ElonginC (17–112) and ElonginB (1–104) were co-transformed into *Escherichia coli* BL21(DE3) and grown at 37 °C. Upon reaching an OD_{600} ~ 1.0, the temperature was lowered to 23 °C and expression was induced using 0.3 mM isopropyl β-D-1-thiogalactopyranoside (IPTG). Sixteen hours post-induction, *E. coli* cells were lysed using a pressure cell homogenizer (Stansted Fluid Power) and the lysate was clarified by centrifugation. His_6-tagged VCB was purified on a HisTrap FF affinity column (GE Healthcare) by elution with an imidazole gradient. The His_6-tag was then removed using TEV protease (overnight, 4 °C) and protein was reapplied to the HisTrap FF column, allowing impurities to bind and the cleaved complex to flowthrough without binding. VCB was then additionally purified by anion exchange and size-exclusion chromatography using Resource Q and Superdex-75 columns (GE Healthcare), respectively. The final purified complex was stored in 20 mM 4-(2-hydroxyethyl)-1-piperazineethanesulfonic acid (HEPES) (pH 7.5) 100 mM sodium chloride and 1 mM TCEP.

$Brd2^{BD1}$ (71–194), $Brd2^{BD2}$ (344-455), $Brd3^{BD1}$ (24-146), $Brd3^{BD2}$ (306-416), $Brd4^{BD1}$ (44-178) and $Brd4^{BD2}$ (333–460) were expressed with an N-terminal His_6 tag in *E. coli* BL21(DE3) at 15 °C for 18 h using 0.3 mM IPTG. Cells were lysed as above, and His_6-tagged BDs were purified on Nickel Sepharose 6 fast flow beads (GE Healthcare) by elution with imidazole. Eluted BDs were then additionally purified by size-exclusion chromatography using a Superdex-75 column. The final purified proteins were stored in 20 mM HEPES (pH 7.5), 100 mM sodium chloride and 1 mM TCEP. All chromatography purification steps were performed using Äkta FPLC purification systems (GE Healthcare) or glass econo-columns (Bio-Rad) at room temperature.

Isothermal titration calorimetry (ITC)

The titrations were all performed as reverse mode (protein in syringe, ligand in cell) and consisted of 19 injections of 2 μl protein solution (20 mM bis–tris propane, 100 mM NaCl, 1 mM tris(2-carboxyethyl)phosphine (TCEP), pH 7.4) at a rate of 0.5 μl/s at 120 s time intervals. An initial

injection (0.4 µl) was made and discarded during data analysis. All experiments were performed at 25 °C, whilst stirring at 600 r.p.m.

PROTAC **1** was diluted from a 10 mM DMSO stock solution to 20 µM in buffer containing 20 mM bis-tris propane, 100 mM NaCl, 1 mM tris(2-carboxyethyl)phosphine (TCEP), pH 7.4. The final DMSO concentration was 0.2 %. Bromodomain (200 µM, in the syringe) was titrated into **1** (20 µM, in the cell). At the end of the titration, the excess of solution was removed from the cell, the syringe was washed and dried, VCB complex (168 µM, in the same buffer) was loaded in the syringe and titrated into the complex PROTAC–bromodomain.

Titrations for the binary complex **1**–VCB were performed as follows: to the solution of **1** (20 µM, in the cell), buffer (38.4 µL) was added by means of a single ITC injection. The excess solution was removed from the cell, the syringe was washed and dried and VCB complex (168 µM, in the same buffer) was loaded in the syringe and titrated into the diluted PROTAC solution. The data were fitted to a single-binding-site model to obtain the stoichiometry n, the dissociation constant K_d and the enthalpy of binding ΔH using the d analyzed using the MicroCal PEAQ-ITC analysis software provided by the manufacturer. Cooperativity (α) values were calculated from the ratio of binary K_d and ternary K_d values determined for VCB binding to **1** or **1**:bromodomain, respectively. The reported values are the mean ± standard deviation from independent measurements.

Fluorescence polarization assay

FP competitive binding assays were run in triplicate on 384-well plates [Corning 3575] as described previously,[8] with all measurements taken using a PHERAstar FS (BMG LABTECH) with fluorescence excitation and emission wavelengths (λ) of 485 and 520 nm, respectively. The final assay volume was 15 µL, with each well solution containing 15 nM VCB protein, 10 nM FAM-labeled HIF-1α peptide (FAM-DEALAHypYIPMDDDFQLRSF, "JC9") and decreasing concentrations of PROTAC (14-point 2-fold serial dilution starting from 50 µM) or PROTAC:bromodomain (14-point 2-fold serial dilutions starting from 10 µM PROTAC:20 µM bromodomain). All components were dissolved from stock solutions using 100 mM Bis-Tris propane, 100 mM NaCl, 1 mM TCEP, pH 7.5, and DMSO was added as appropriate to ensure a final concentration of 1%. Control wells containing VCB and JC9 with no compound (zero displacement), or JC9 in the absence of protein (maximum displacement) were also included. Data were normalized to control values in order to obtain percentage displacements, which were then plotted against Log[PROTAC]. Curves were fitted by nonlinear regression using Prism (v. 8.0.1, GraphPad) to determine the IC_{50} values for each titration. K_i values were back-calculated from the K_d for JC9 (~2 nM, determined from direct binding) and fitted IC_{50} values, as described previously. Cooperativity (α) values were calculated from the ratio of binary K_i and ternary K_i values determined for JC9 displacement by 1 alone or 1 + bromodomain, respectively.

Crystallography

The ternary complex VCB:**1**:Brd4^{BD2} was prepared by combining VCB, Brd4^{BD2}, and **1** in a 1:1:1 molar ratio and incubating for 15 min at RT. Crystals were grown at 20 °C using the hanging-drop diffusion method by mixing equal volumes of ternary complex solution and a crystallization solution containing 10% (w/v) PEG 8000, 0.1 M Tris-HCl (pH 7.5) and 0.1 M $MgCl_2$. Crystals appeared almost immediately, and were ready for harvest within 24 h. Crystals were flash-frozen in liquid nitrogen using 20% (v/v) ethylene glycol in liquor solution as a cryoprotectant. Diffraction data were collected at Diamond Light Source beamline I04 using a Pilatus 6M-F detector at a wavelength of 0.9750 Å. Reflections were indexed and integrated using XDS [9], and scaling and merging were performed with AIMLESS [10] in CCP4i.[11] The crystals belonged to space group $P3_2$, and there were two copies of the ternary complex in the asymmetric unit. The

structure was solved by molecular replacement using PHASER MR [12] and search models derived from the coordinates for the VCB:MZ1:Brd4^{BD2} ternary complex (PDB entry 5T35). The initial model underwent iterative rounds of model building and refinement with COOT [13] and REFMAC5,[14] respectively. All riding hydrogens were excluded from the output coordinate files but included for refinement. Compound **1** geometry restraints for refinement were prepared with the PRODRG [15] server and optimized using eLBOW [16] from the PHENIX suite.[17] Model geometry and steric clashes were validated using the MOLPROBITY server.[18] Ramachandran plots indicate that 96.8% of backbone torsion angles are in the favored region and there are no outliers. The structure has been deposited in the protein data bank (PDB) with accession code 6SIS; data collection and refinement statistics are presented in table S2. Interfaces observed in the crystal structure were calculated using PISA, and all figures were generated using PyMOL.

	VCB:**1**:Brd4^{BD2}
**Data Collection**	
Space Group	*P3$_2$*
Cell Dimensions	
a, b, c (Å)	99.5 99.5 148.4
α, β, γ, (°)	90 90 120
Resolution (*Å*)	49.7 – 3.50 (3.83 – 3.50)*
No. unique reflections	19215 (4396)
R_{merge} (%)	13.5 (58.7)
I/σ(I)	6.3 (1.5)
$CC_{1/2}$	0.984 (0.542)
Completeness (%)	92.6 (88.4)
Redundancy	2.6 (2.6)
**Refinement**	
Resolution	49.7 – 3.5
R_{work} / R_{free}	22.0 / 24.7
No. atoms	
Protein	7208
Ligand	154
Water	7
B factors	
Protein	112
Ligand	90
Water	37
R.m.s. deviations	
Bond lengths (Å)	0.005
Bond angles (°)	0.729
Buried surface area (Å).	
VHL:Brd4^{BD2}	661
VHL:**1**	961
Brd4^{BD2}:**1**	1064

Table S2 *Crystallographic data collection and refinement statistics. * Values in parentheses are for highest-resolution shell.*

Tissue culture

HeLa cells were kept in DMEM (Gibco) supplemented with 10%(v/v) FBS (Gibco), 2 mM L-glutamine (Gibco), penicillin (100 units /mL) and streptomycin (100 µg/mL) (Gibco). Mv4;11 and 22RV1 (ATCC CRL-2505) cells were kept in RPMI 1640 supplemented with 10% (v/v) FBS (Gibco), 2 mM L-glutamine (Gibco), penicillin (100 units /mL) and streptomycin (100 µg/mL) (Gibco). Cells were kept at 37 °C, 5% CO_2.

Testing compounds in cells

HeLa cells or 22RV1 cells (6×10^5) were seeded onto each well on a six-well plate 24 h before treatment with test compounds. To treat the cells with the test compounds, medium was replaced with fresh medium containing specified concentration of test compounds for either 4 h or 18 h. The same DMSO concentration was maintained in all samples. At the end of treatment, cells were washed with PBS and then lysed in RIPA buffer (Sigma, R0278) supplemented with HALT Protease inhibitor (ThermoFisher, # 78842). Lysate was incubated on ice for 15 min and then clarified by centrifugation (20,000 × *g*, 10 min, 4 °C). Supernatant was collected and protein concentration was determined by BCA assay. The rest of the clarified lysate was kept at -20°C before further processing.

Immunoblotting

Protein on gel was transferred to nitrocellulose membrane using the Bio-Rad Transblot system according to manufacturer guidelines. Blots were probed with anti-Brd4 (AbCam, ab128874), anti-Brd3 (AbCam, ab50818), anti-Brd2 (AbCam, ab139690) and anti-c-MYC (Y69 ab32072) followed by hFAB™ Rhodamine Anti-Tubulin (Biorad #12004165) and anti-mouse IgG (Licor, 926-32210) or anti-rabbit IgG (Licor, 926-32213) antibodies and bands visualized using Bio-Rad ChemiDoc MP Imaging System.

Western Blot Quantification

Image processing and band intensity quantification were performed using Bio-Rad Image Lab

software version 6.0.0. Reported band intensities are first normalized to tubulin loading control and then to DMSO only treated sample.

Cell Viability Assay

MV4;11 or 22RV1 cells (15000 or 5000 cells per well respectively) were dosed for 48 or 72 h with compounds serially diluted (1:3) on a clear-bottom 384-well plate with a final concentration of 0.05% (v/v) DMSO. After treatment, Promega CellTiter-Glo luminescent cell viability assay (G7572) was added to the cells according to the manufacturer instructions. The signal from each well was recorded on a BMG Labtech Pherastar luminescence plate reader with recommended settings. Normalized data were analyzed and plotted with Graphpad Prism software in the 'log(inhibitor) vs. response -- Variable slope (four parameters)' module. EC50 values of each test compound were derived from this plot.

	MZ1	**1**	cis-MZ1
MV4;11 EC50 (nM)<br>48 h	149	304	N/A
MV4;11 EC50 (nM)<br>72 h	7	47	1420
22RV1 EC50 (nM)<br>72 h	191	643	1457

Copies of NMR spectra

Compound **5**, ^{1}H-NMR $CDCl_3$, 500 MHz

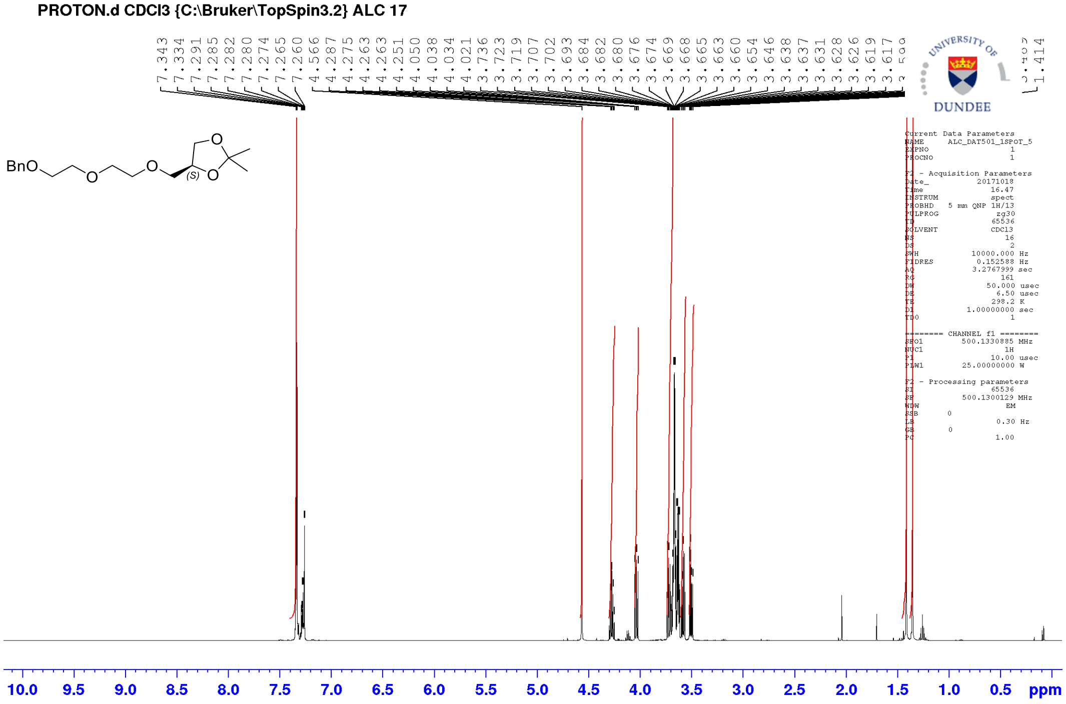

Compound **5**, ^{13}C-NMR $CDCl_3$, 126 MHz

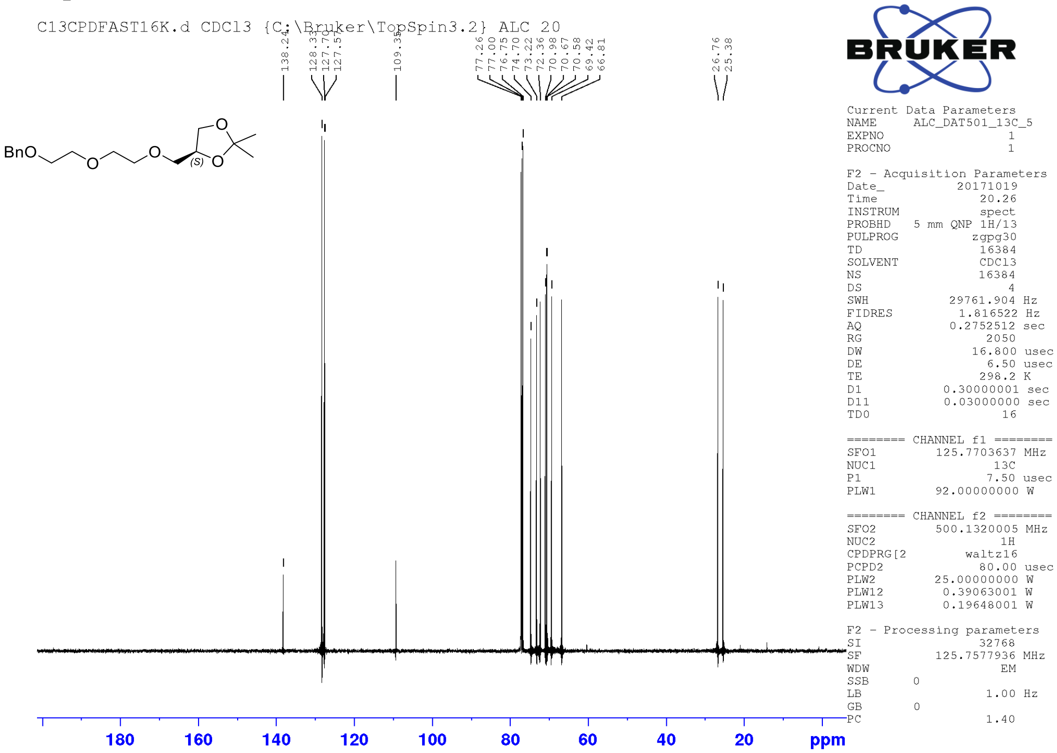

Compound **6**, ^{1}H-NMR $CDCl_3$, 500 MHz

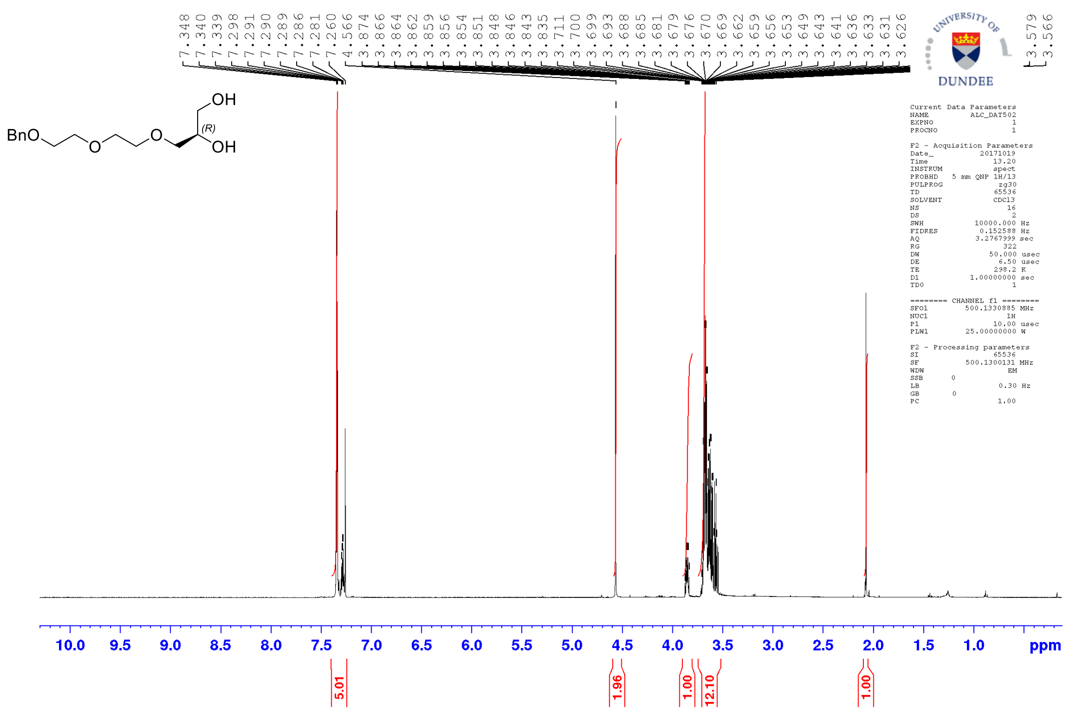

Compound **6**, ^{13}C-NMR $CDCl_3$, 126 MHz

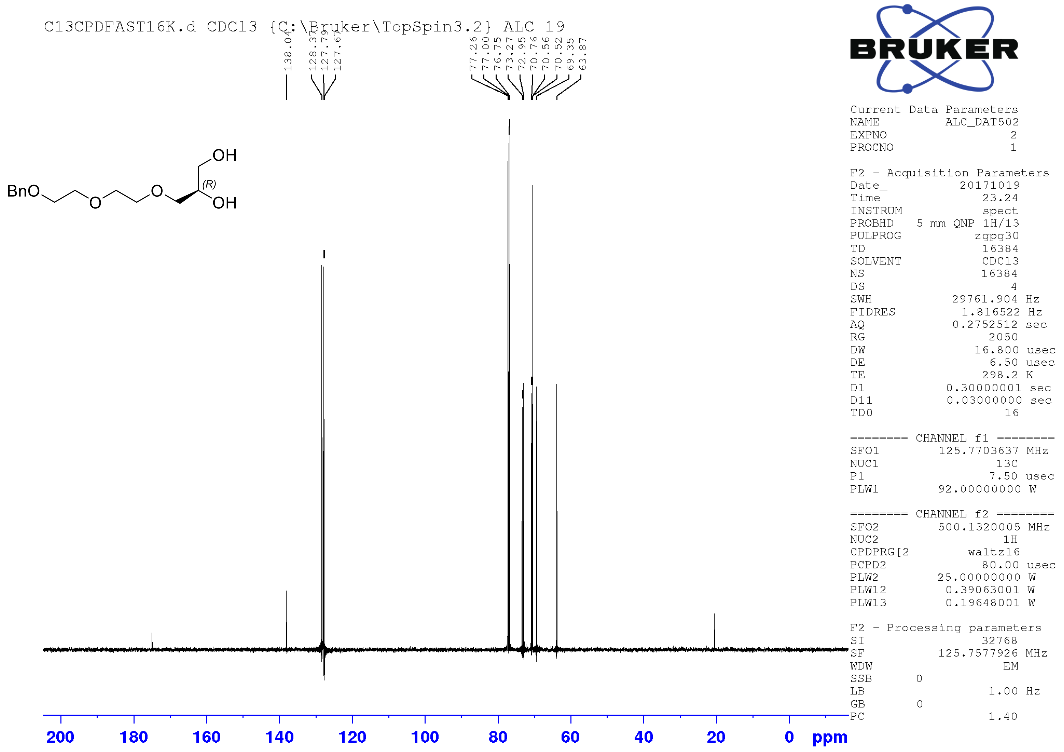

Compound **7**, ^{1}H-NMR $CDCl_3$, 500 MHz

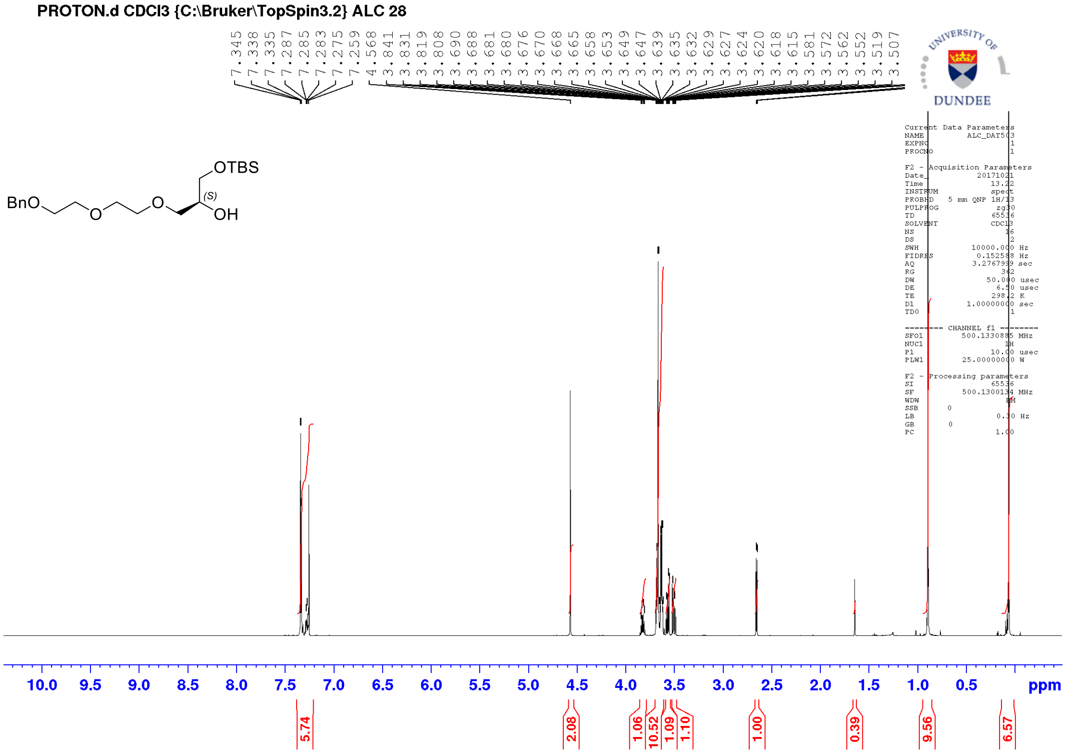

Compound **7**, ^{13}C-NMR $CDCl_3$, 126 MHz

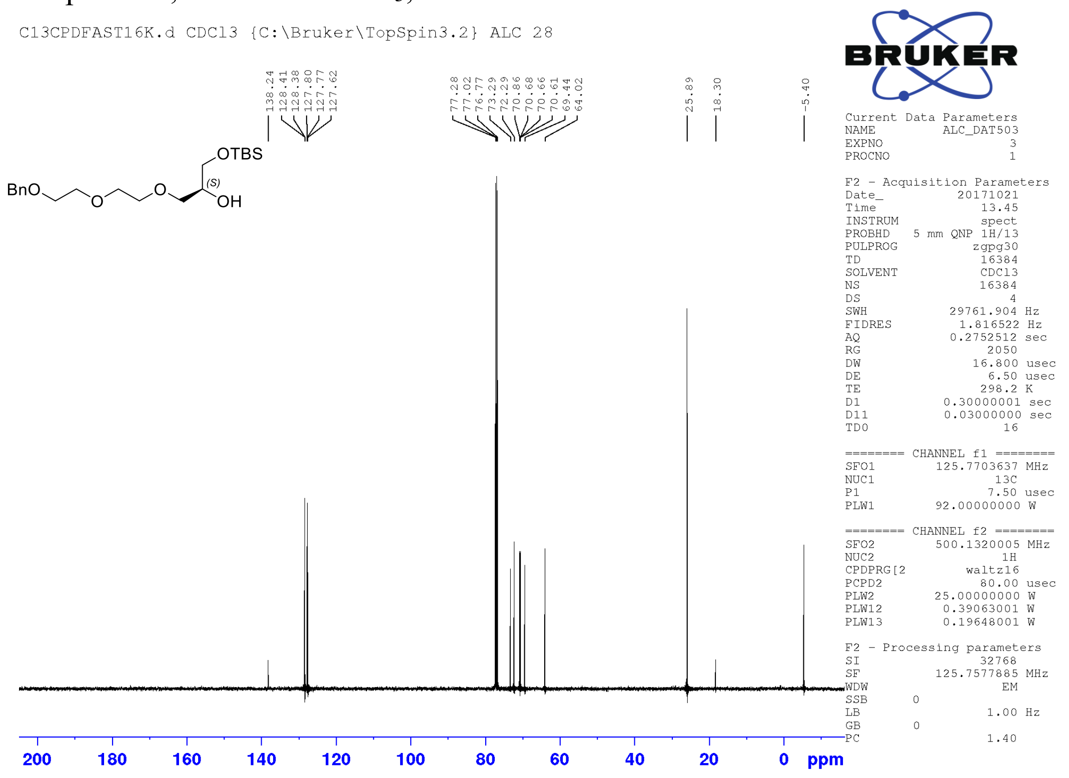

Compound **8**, ^{1}H-NMR $CDCl_3$, 400 MHz

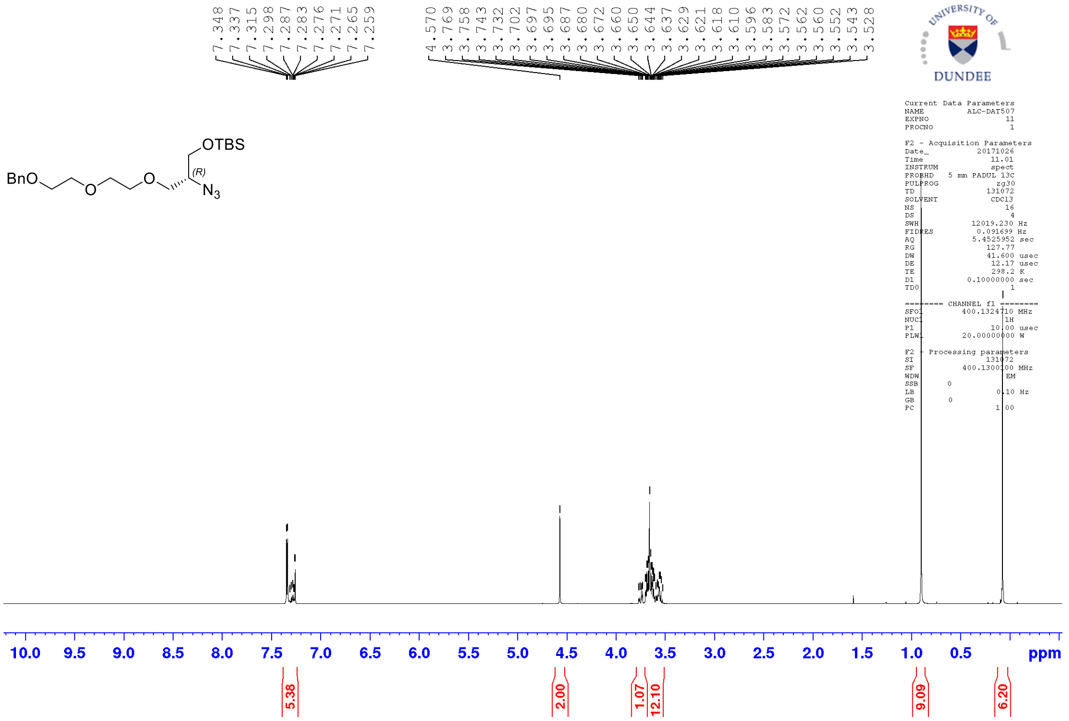

Compound **8**, ^{13}C-NMR $CDCl_3$, 101 MHz

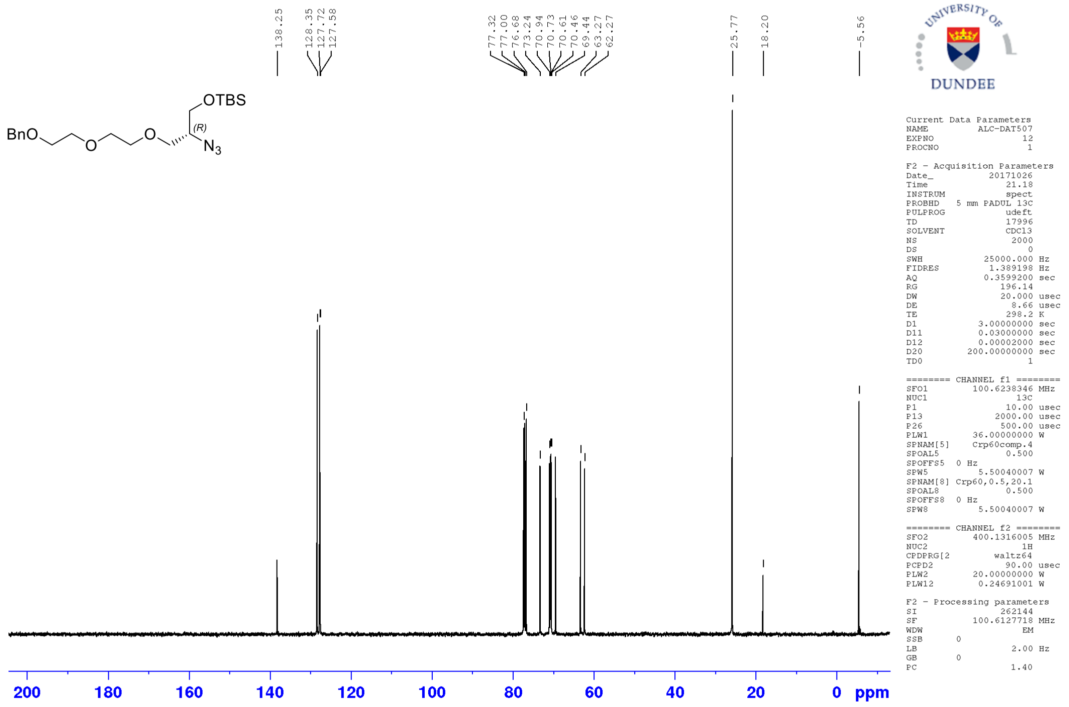

Compound **10**, ^{1}H-NMR $CDCl_3$, 400 MHz

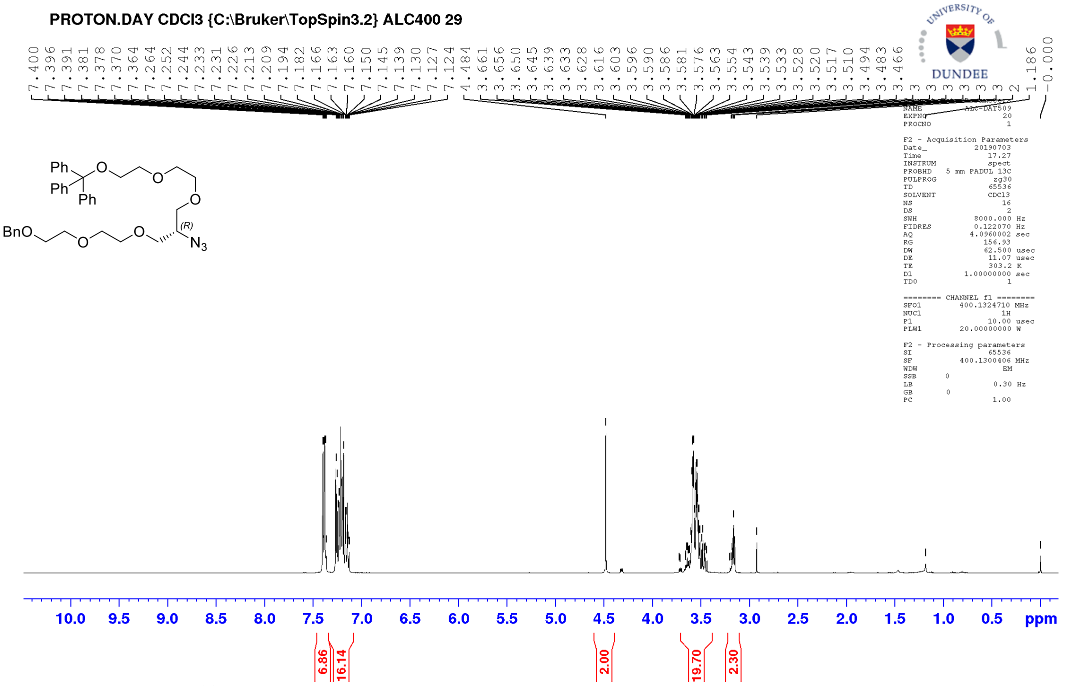

Compound **10**, ^{13}C-NMR $CDCl_3$, 101 MHz

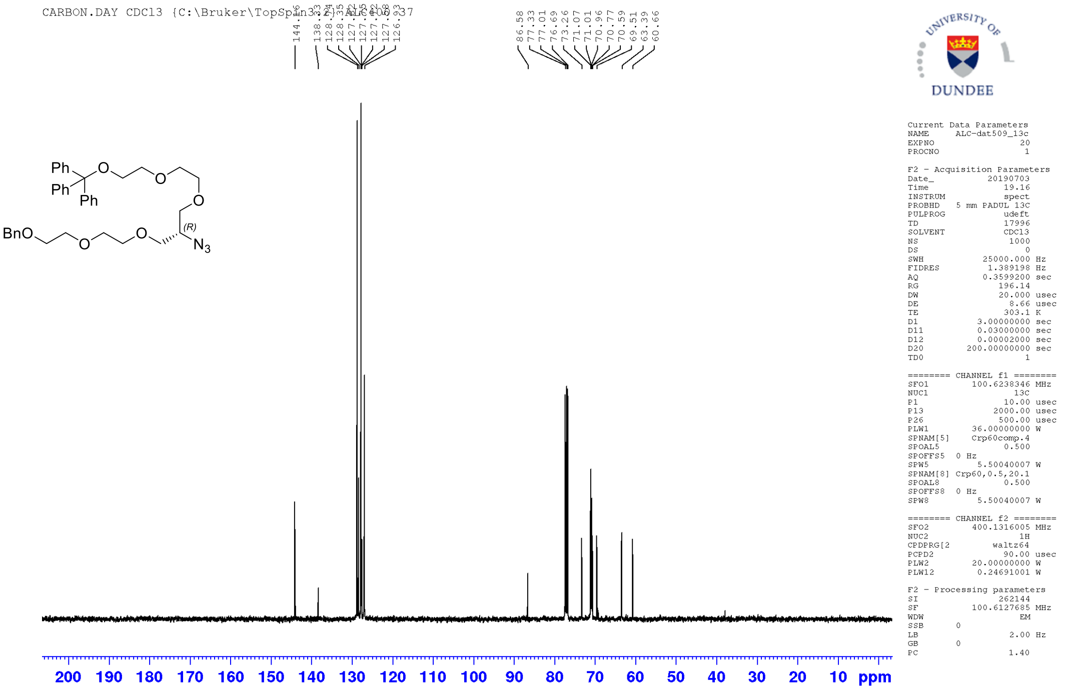

Compound **11**, ^{1}H-NMR $CDCl_3$, 500 MHz

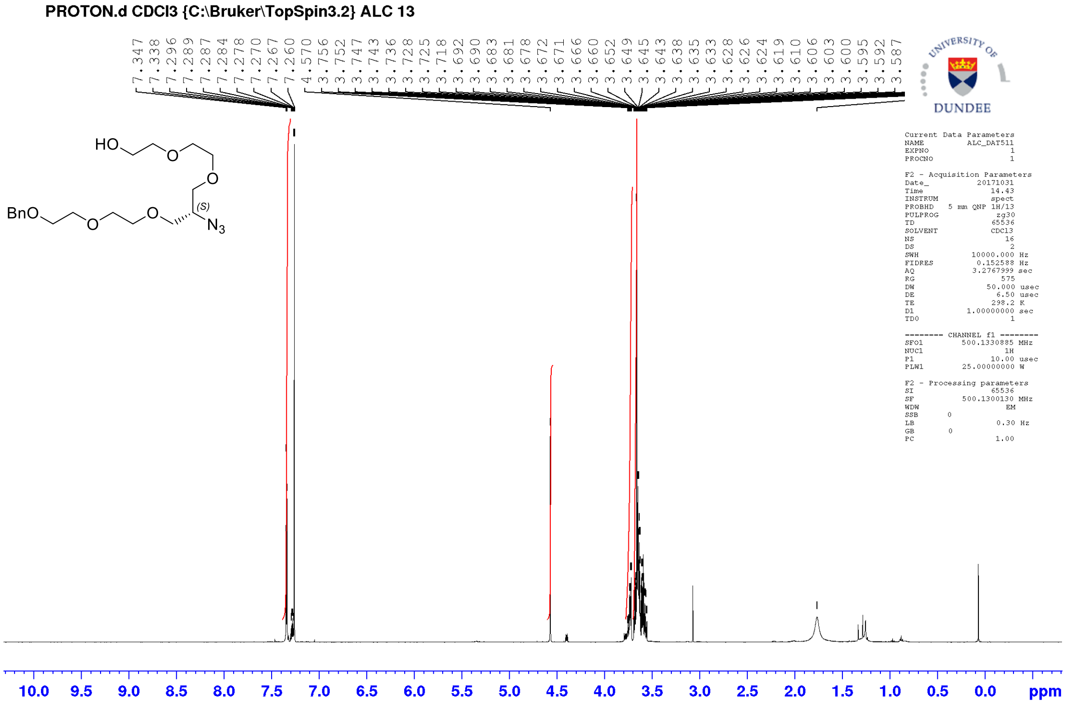

Compound **11**, ^{13}C-NMR $CDCl_3$, 101 MHz

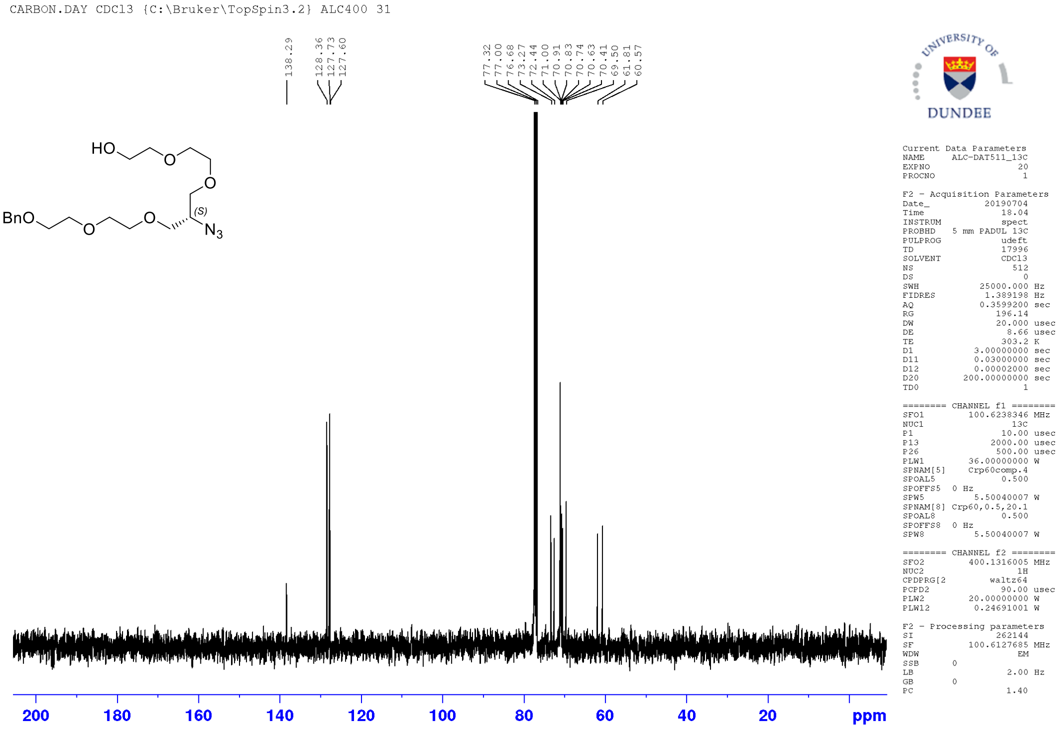

Compound **12**, ^{1}H-NMR $CDCl_3$, 500 MHz

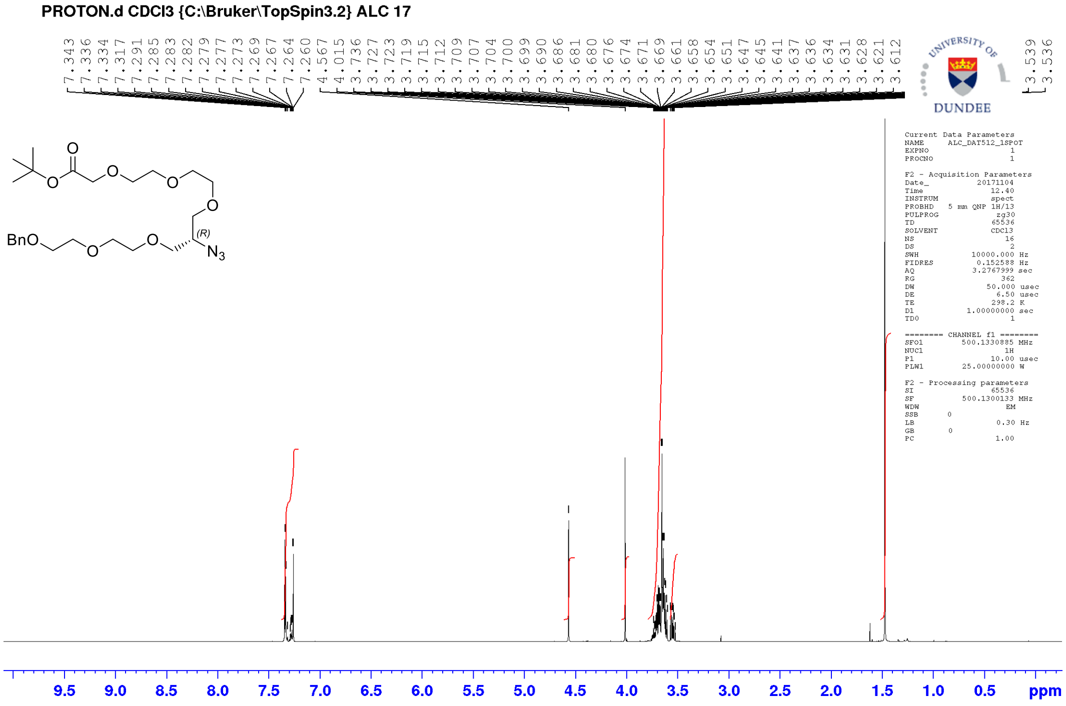

Compound **12**, ^{13}C-NMR $CDCl_3$, 126 MHz

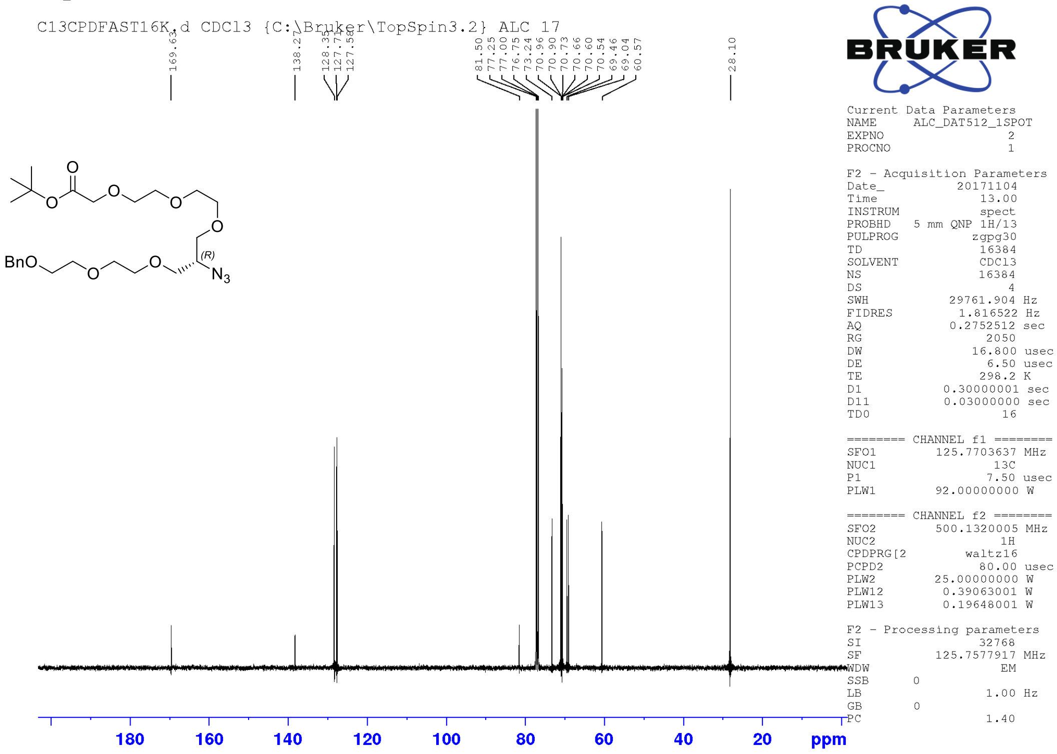

Compound **13**, ^{1}H-NMR $CDCl_3$, 500 MHz

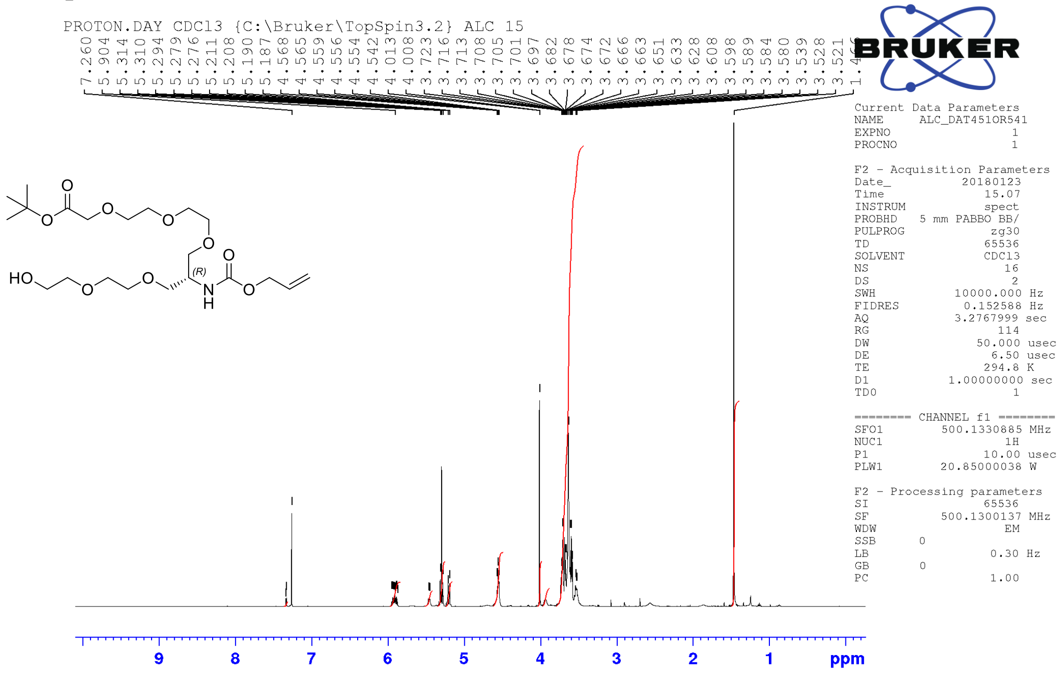

Compound **13**, ^{13}C-NMR $CDCl_3$, 101 MHz

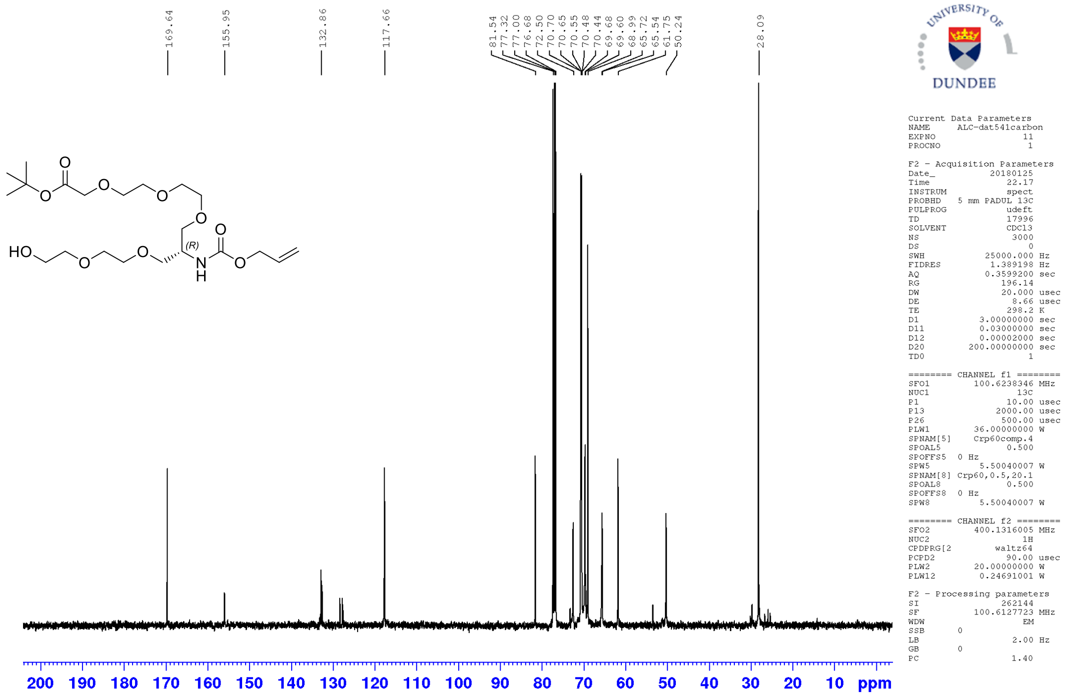

Compound **16**, ^{1}H-NMR MeOD, 400 MHz

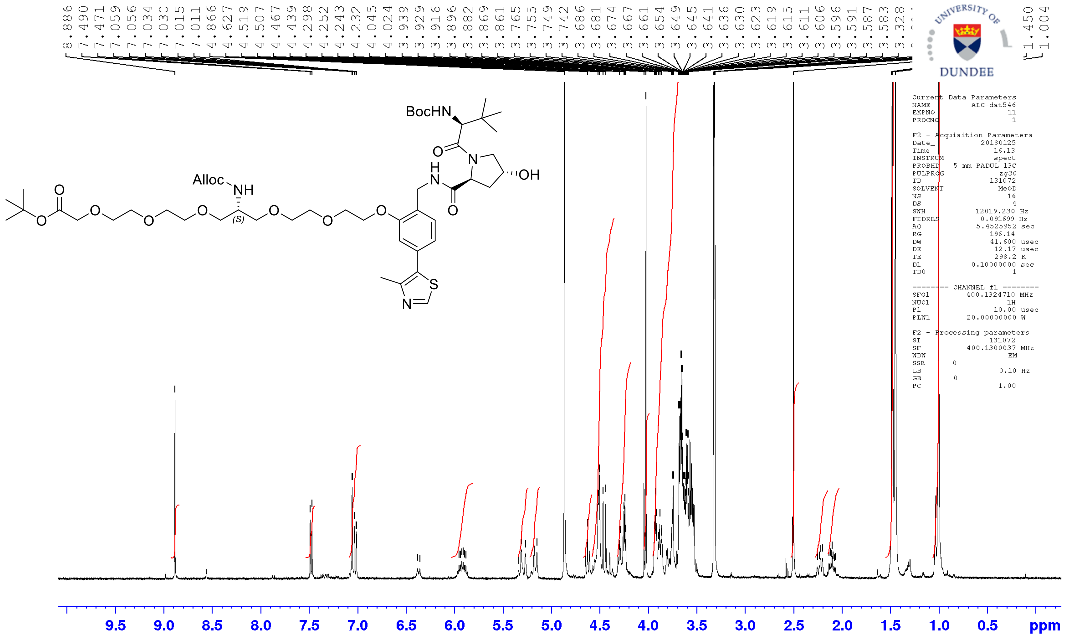

Compound **16**, ^{13}C-NMR MeOD, 101 MHz

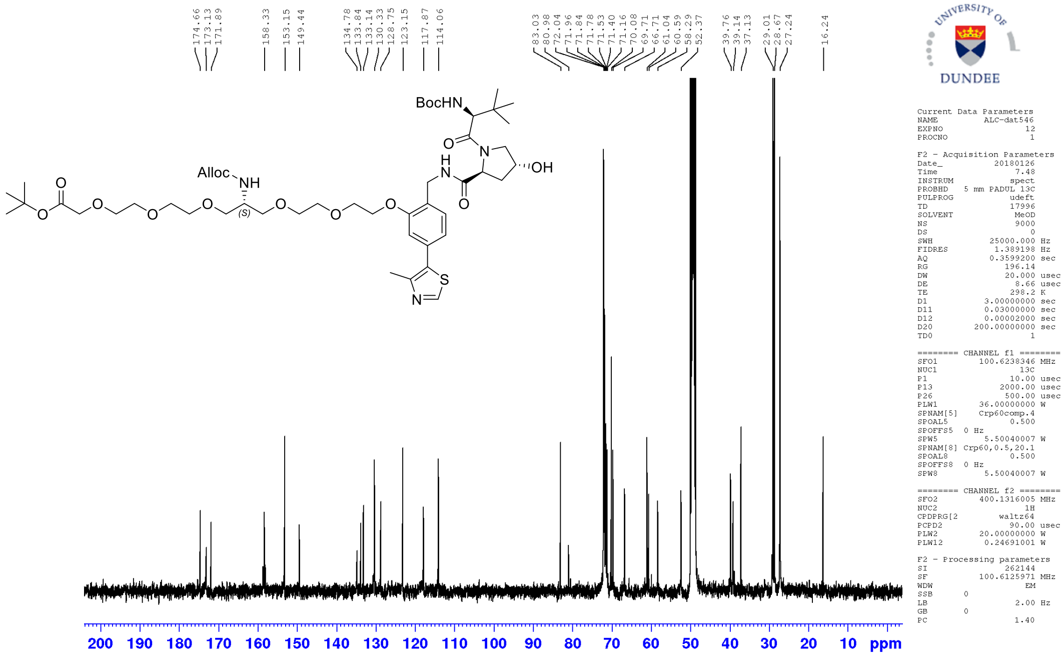

Compound **17**, ^{1}H-NMR MeOD, 400 MHz

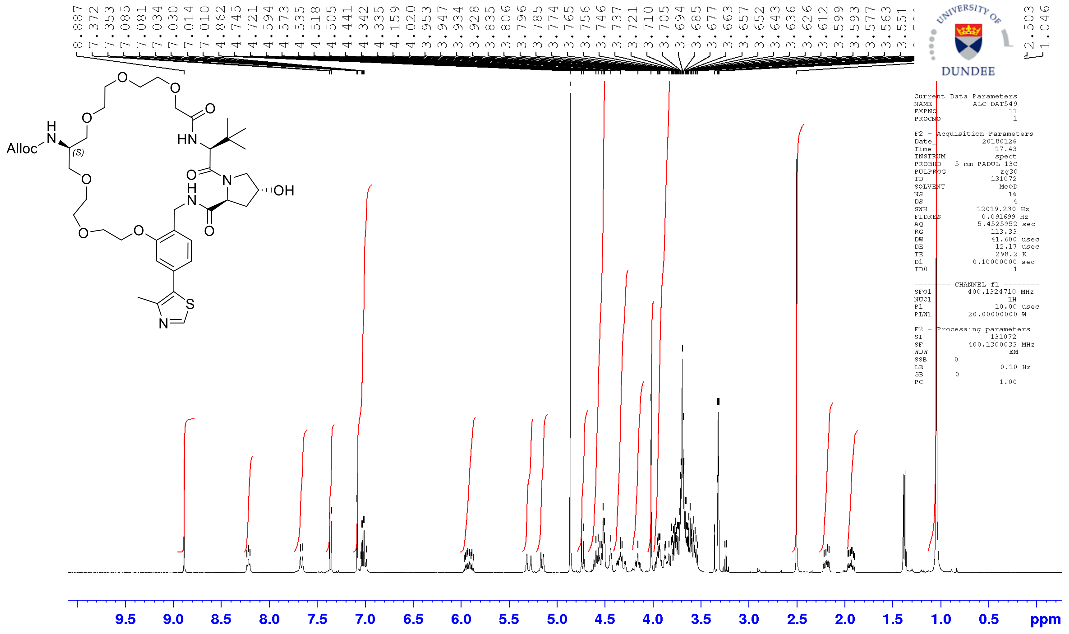

Compound **17**, ^{13}C-NMR MeOD, 101 MHz

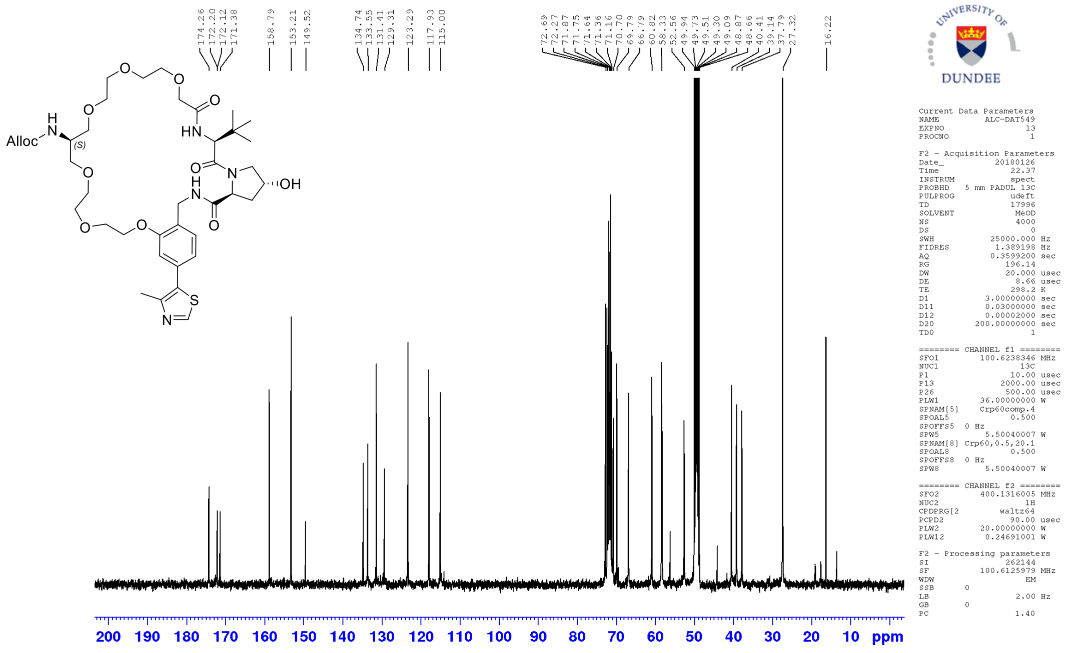

Compound **18**, ^{1}H-NMR DMSO-D6, 400 MHz

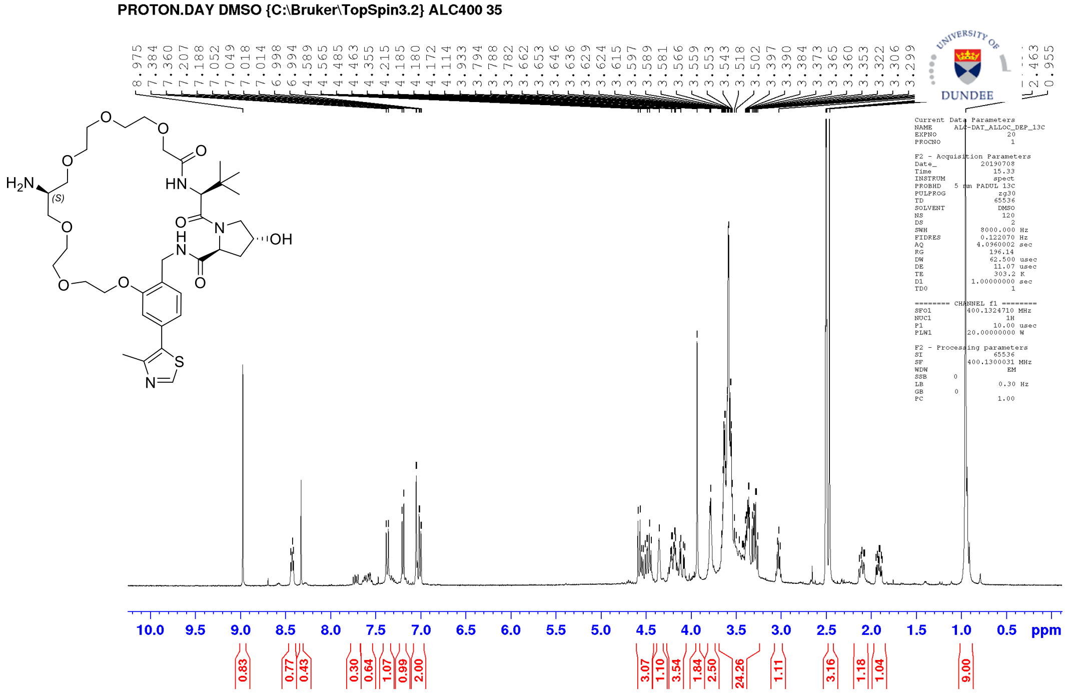

Compound **17**, ^{13}C-NMR DMSO-D6, 101 MHz

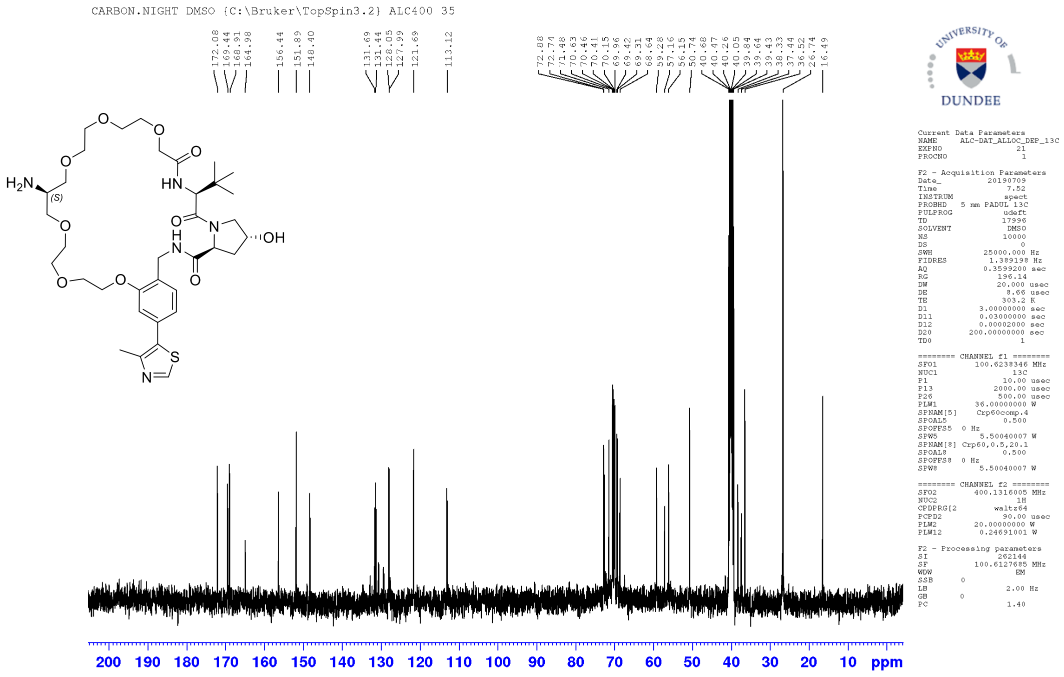

Compound **1**, ^{1}H-NMR MeOD, 400 MHz

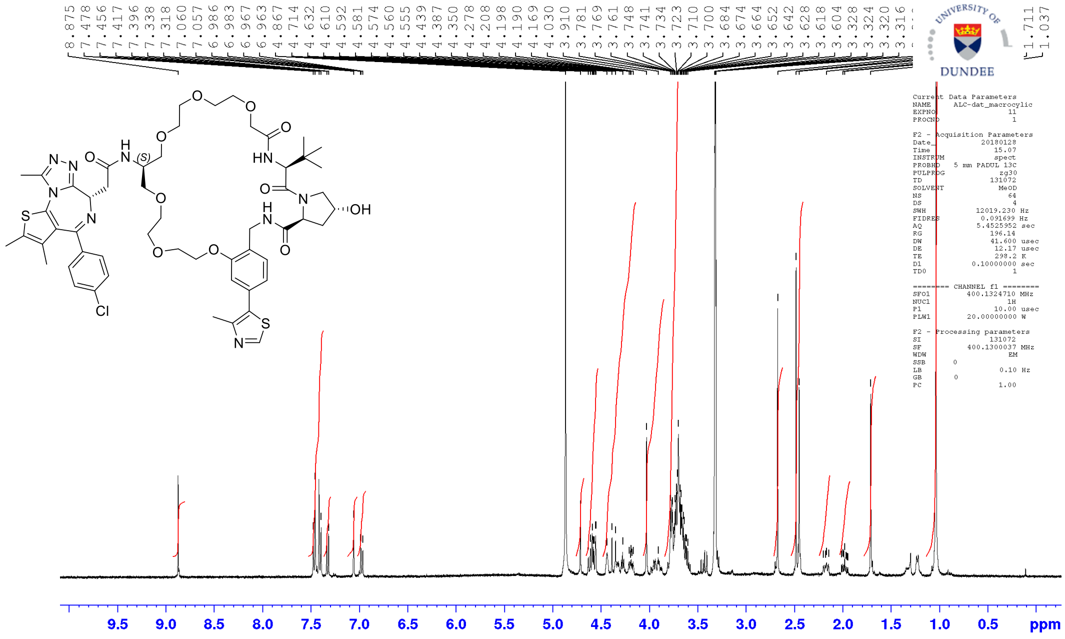

Compound **1**, ^{13}C-NMR MeOD, 101 MHz

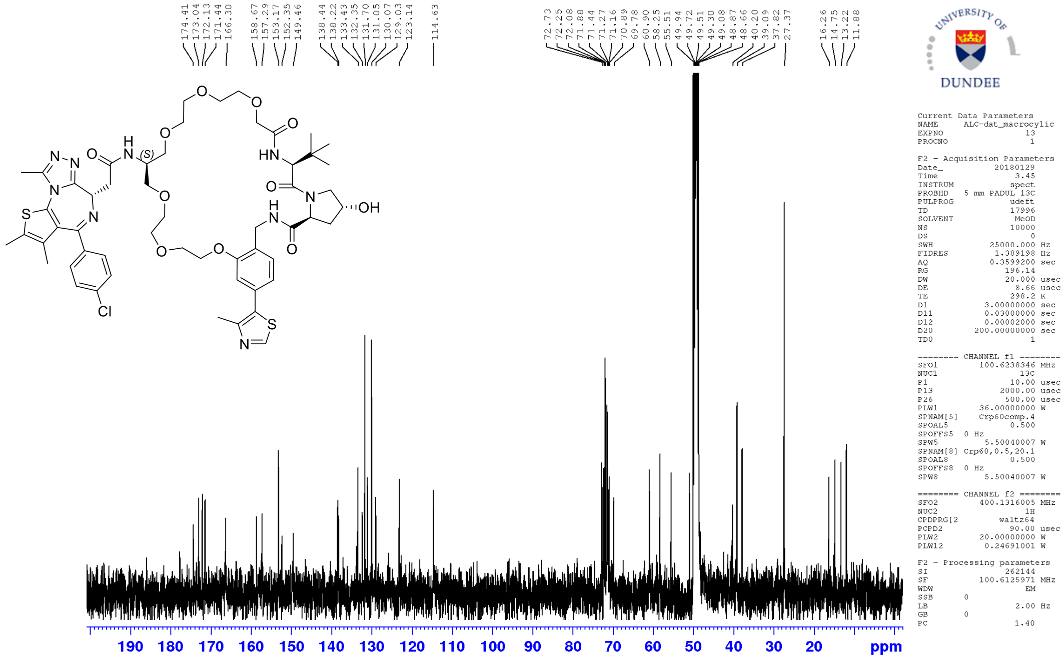

Supplementary References

[1] M. S. Gadd, A. Testa, X. Lucas, K.-H. Chan, W. Chen, D. J. Lamont, M. Zengerle, A. Ciulli, *Nat. Chem. Biol.* **2017**, *13*, 514–521.

[2] C. R. Søndergaard, M. H. M. Olsson, M. Rostkowski, J. H. Jensen, *J. Chem. Theory Comput.* **2011**, *7*, 2284–2295.

[3] E. Harder, W. Damm, J. Maple, C. Wu, M. Reboul, J. Y. Xiang, L. Wang, D. Lupyan, M. K. Dahlgren, J. L. Knight, et al., *J. Chem. Theory Comput.* **2016**, *12*, 281–296.

[4] W. Humphrey, A. Dalke, K. Schulten, *J.Mol.Graphics* **1996**, *14*, 33–8– 27–8.

[5] S. Kawamura, Y. Ito, T. Hirokawa, E. Hikiyama, S. Yamada, S. Shuto, *J. Med. Chem.* **2018**, *61*, 4020–4029.

[6] V. Zoppi, S. J. Hughes, C. Maniaci, A. Testa, T. Gmaschitz, C. Wieshofer, M. Koegl, K. M. Riching, D. L. Daniels, A. Spallarossa, et al., *J. Med. Chem.* **2019**, *62*, 699–726.

[7] M. Zengerle, K.-H. Chan, A. Ciulli, *ACS Chem. Biol.* **2015**, *10*, 1770–1777.

[8] M. J. Roy, S. Winkler, S. J. Hughes, C. Whitworth, M. Galant, W. Farnaby, K. Rumpel, A. Ciulli, *ACS Chem. Biol.* **2019**, *14*, 361–368.

[9] W. Kabsch, *Acta Crystallogr D Biol Crystallogr* **2010**, *66*, 125–132.

[10] P. Evans, *Acta Crystallogr D Biol Crystallogr* **2006**, *62*, 72–82.

[11] M. D. Winn, C. C. Ballard, K. D. Cowtan, E. J. Dodson, P. Emsley, P. R. Evans, R. M. Keegan, E. B. Krissinel, A. G. W. Leslie, A. McCoy, et al., *Acta Crystallogr D Biol Crystallogr* **2011**, *67*, 235–242.

[12] A. J. McCoy, R. W. Grosse-Kunstleve, P. D. Adams, M. D. Winn, L. C. Storoni, R. J. Read, *J Appl Crystallogr* **2007**, *40*, 658–674.

[13] P. Emsley, K. Cowtan, *Acta Crystallogr D Biol Crystallogr* **2004**, *60*, 2126–2132.

[14] G. N. Murshudov, A. A. Vagin, E. J. Dodson, *Acta Crystallogr D Biol Crystallogr* **1997**, *53*, 240–255.

[15] A. W. Schüttelkopf, D. M. F. van Aalten, *Acta Crystallogr D Biol Crystallogr* **2004**, *60*, 1355–1363.

[16] N. W. Moriarty, R. W. Grosse-Kunstleve, P. D. Adams, *Acta Crystallogr D Biol Crystallogr* **2009**, *65*, 1074–1080.

[17] P. D. Adams, P. V. Afonine, G. Bunkoczi, V. B. Chen, I. W. Davis, N. Echols, J. J. Headd, L.-W. Hung, G. J. Kapral, R. W. Grosse-Kunstleve, et al., *Acta Crystallogr D Biol Crystallogr* **2010**, *66*, 213–221.

[18] V. B. Chen, W. B. Arendall, J. J. Headd, D. A. Keedy, R. M. Immormino, G. J. Kapral, L. W. Murray, J. S. Richardson, D. C. Richardson, *Acta Crystallogr D Biol Crystallogr* **2010**, *66*, 12–21.
